# Dendritic cell–intrinsic LKB1-AMPK/SIK signaling controls metabolic homeostasis by limiting the hepatic Th17 response during obesity

**DOI:** 10.1172/jci.insight.157948

**Published:** 2023-06-08

**Authors:** Hendrik J.P. vanderZande, Eline C. Brombacher, Joost M. Lambooij, Leonard R. Pelgrom, Anna Zawistowska-Deniziak, Thiago A. Patente, Graham A. Heieis, Frank Otto, Arifa Ozir-Fazalalikhan, Maria Yazdanbakhsh, Bart Everts, Bruno Guigas

**Affiliations:** 1 Department of Parasitology, Leiden University Medical Center (LUMC), Leiden, the Netherlands.; 2 Department of Parasitology and; 3 Department of Immunology, Institute of Functional Biology and Ecolog, Faculty of Biology, University of Warsaw, Warsaw, Poland.

**Keywords:** Immunology, Metabolism, Dendritic cells, Obesity, T cells

## Abstract

Obesity-associated metabolic inflammation drives the development of insulin resistance and type 2 diabetes, notably through modulating innate and adaptive immune cells in metabolic organs. The nutrient sensor liver kinase B1 (LKB1) has recently been shown to control cellular metabolism and T cell priming functions of DCs. Here, we report that hepatic DCs from high-fat diet–fed (HFD-fed) obese mice display increased LKB1 phosphorylation and that LKB1 deficiency in DCs (CD11c^ΔLKB1^) worsened HFD-driven hepatic steatosis and impaired glucose homeostasis. Loss of LKB1 in DCs was associated with increased expression of Th17-polarizing cytokines and accumulation of hepatic IL-17A^+^ Th cells in HFD-fed mice. Importantly, IL-17A neutralization rescued metabolic perturbations in HFD-fed CD11c^ΔLKB1^ mice. Mechanistically, deficiency of the canonical LKB1 target AMPK in HFD-fed CD11c^ΔAMPKα1^ mice recapitulated neither the hepatic Th17 phenotype nor the disrupted metabolic homeostasis, suggesting the involvement of other and/or additional LKB1 downstream effectors. We indeed provide evidence that the control of Th17 responses by DCs via LKB1 is actually dependent on both AMPKα1 and salt-inducible kinase signaling. Altogether, our data reveal a key role for LKB1 signaling in DCs in protection against obesity-induced metabolic dysfunctions by limiting hepatic Th17 responses.

## Graphical abstract

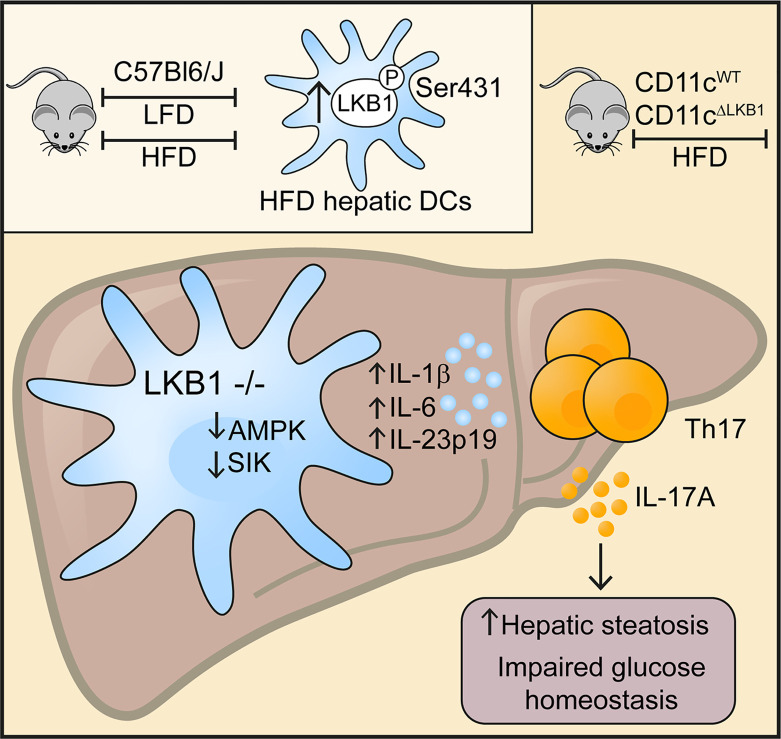

## Introduction

Obesity is associated with chronic low-grade inflammation, also known as metabolic inflammation, where continuous overnutrition generates a self-sustained inflammatory loop in metabolic tissues that drives insulin resistance and type 2 diabetes (1). One of the hallmarks of metabolic inflammation is the accumulation of myeloid cells in the main metabolic organs — i.e., white adipose tissue (WAT), liver, and skeletal muscle (2). Macrophage-related cytokines such as TNF and IL-1β were shown to inhibit insulin signaling (3, 4); therefore, macrophages are considered key players in the etiology of tissue-specific insulin resistance. However, DCs also accumulate in WAT and liver during obesity and are associated with metabolic dysfunctions. Indeed, depletion of the entire DC population or specific conventional DC (cDC) subsets in different genetic mouse models alleviates adipose tissue and/or hepatic inflammation, although the underlying mechanisms are incompletely understood (5–8).

DCs are specialized antigen-presenting cells that govern T cell responses depending on the inflammatory and metabolic microenvironment. Moreover, modulation of Th cell subsets in metabolic tissues has been shown to play a role in the control of immunometabolic homeostasis. For instance, Th2 cells and Tregs are enriched in lean, insulin-sensitive WAT and contribute to maintenance of tissue-specific insulin sensitivity (9–11). On the contrary, Th17 cells accumulate in WAT and liver during obesity and are associated with hepatic steatosis and insulin resistance (12–16). In addition, preventing CXCR3-dependent hepatic Th17 accrual and blocking IL-17A signaling using neutralizing antibodies both alleviated nonalcoholic fatty liver disease (NAFLD) (15, 16), suggesting an important contribution of hepatic Th17 cells to NAFLD severity. Although both DCs and Th cell subsets in metabolic tissues have been associated with control of metabolic homeostasis, little is known about the regulation of DC-mediated Th cell polarization in these organs during the development of obesity or its impact on whole-body insulin sensitivity.

DC-mediated priming of Tregs and effector Th1, Th2, and Th17 cells is considered to be driven by metabolic rewiring of DCs in response to environmental cues, and this rewiring controls costimulatory molecule and cytokine expression that shape Th cell polarization (17). For example, in vitro TLR-activated mature DCs depend on glycolysis for fueling their anabolic demands, whereas quiescent DCs mainly rely on fatty acid (FA) oxidation and mitochondrial oxidative phosphorylation (18). As such, the obesity-induced changes in the metabolic organ microenvironment in which DCs reside may impact their T cell–polarizing capacities and contribute to metabolic inflammation (19).

Among the bioenergetic sensors that regulate DC intrinsic metabolism and function in vivo, liver kinase B1 (LKB1) has recently received considerable attention (20–22). The tumor suppressor LKB1 is a serine/threonine kinase that can phosphorylate and activate AMP-activated protein kinase (AMPK) and 12 other members of the AMPK-related family of protein kinases (23, 24), thereby controlling cell growth, survival, polarity, and metabolism (25). In DCs, LKB1 was shown to be a critical regulator of effector T cell and Treg priming, thereby maintaining antitumor immunity (21, 22). We therefore hypothesized that LKB1 in DCs may connect the changing metabolic microenvironment during obesity to altered T cell priming, thereby impacting whole-body metabolic homeostasis.

In the present study, we investigated the role of LKB1 signaling in DC-mediated Th cell priming in metabolic tissues and its impact on metabolic homeostasis. We demonstrate that obesity increased LKB1 phosphorylation in hepatic DCs and that loss of LKB1 in DCs exacerbated hepatic steatosis and impaired glucose homeostasis by promoting hepatic Th17 responses in obese mice. Finally, we demonstrate that LKB1 limits DC-mediated Th17 responses through both AMPKα1 and salt-inducible kinases (SIK).

## Results

### Obesity induces DC activation in metabolic tissues and increases LKB1 phosphorylation in hepatic DCs.

**Figure 1:**
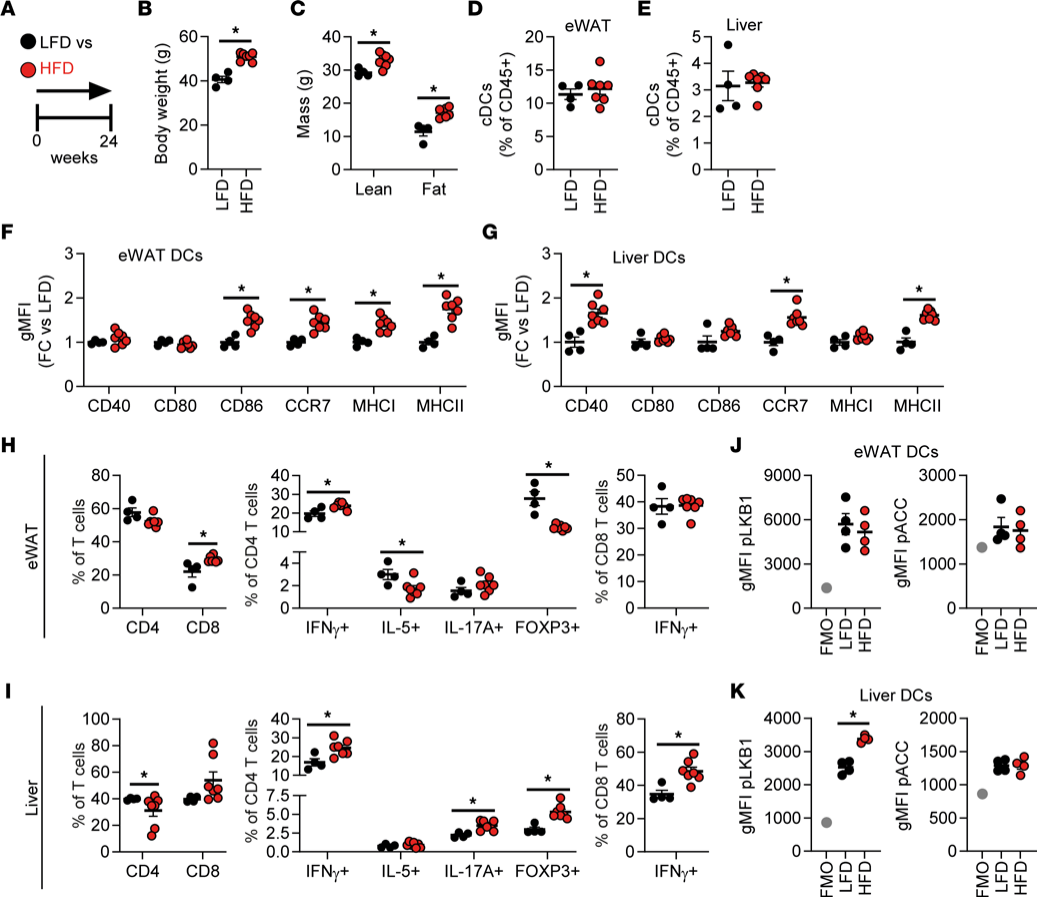
WAT and liver DCs are activated in obese mice. (**A**) Mice were fed a low-fat diet (LFD; black symbols) or a high-fat diet (HFD; red symbols) for 24 weeks. (**B** and **C**) Body weight (**B**) and body composition (**C**) were measured at the end of the experiment. (**D**–**G**) At sacrifice, epidydimal white adipose tissue (eWAT) and liver were collected and immune cells were isolated and analyzed by flow cytometry. Frequencies of DCs within total leukocytes in eWAT (**D**) and liver (**E**). Relative expression of indicated DC markers by eWAT (**F**) and liver DCs (**G**). (**H** and **I**) Cells were restimulated with PMA/ionomycin in the presence of Brefeldin A for detection of intracellular cytokines and were analyzed by flow cytometry. CD4^+^ and CD8^+^ T cell, IFN-γ^+^ (Th1), IL-5^+^ (Th2), IL-17A^+^ (Th17), and FOXP3^+^ (Treg) CD4^+^ T cell and IFN-γ^+^CD8^+^ T cell percentages in eWAT (**H**) and liver (**I**). (**J** and **K**) eWAT and liver were immediately formaldehyde fixed after collection, and immune cells were isolated. Phosphorylated LKB1 (Ser431) and ACC (Ser79) were measured in DCs from eWAT (**J**) and liver (**K**) by flow cytometry. Full gating strategies are shown in Supplemental Figure 1. Data are expressed as mean ± SEM. Statistical analyses were performed using unpaired *t* tests. *P < 0.05 versus LFD (*n* = 4–7 mice per group).

To investigate the role of DCs in whole-body metabolic homeostasis during obesity, male C57BL/6J mice were fed a high-fat diet (HFD) for 24 weeks, resulting in significant increases in body weight and fat mass when compared with low-fat diet–fed (LFD-fed) control mice (Figure 1, A–C). Using flow cytometry (Supplemental Figure 1; supplemental material available online with this article; https://doi.org/10.1172/jci.insight.157948DS1), we assessed the frequency and phenotype of DCs in metabolic tissues from lean and obese mice. The number of leukocytes per g tissue was found to be significantly increased in WAT but not in the liver from obese mice, likely due to increased liver weight (Supplemental Figure 2, A and B). Although DC abundance was not affected (Figure 1, D and E), DCs from both tissues exhibited increased expression of activation markers (Figure 1, F and G), a feature specific to metabolic tissues, since activation status of DCs remained largely unchanged in the spleen (Supplemental Figure 2C). These changes in DC phenotypes were associated with alterations in the Th cell pool in metabolic tissues. In eWAT, IFN-γ^+^ Th1 cells were increased at the expense of IL-5^+^ Th2 cells and FOXP3^+^ Tregs, while in the liver, we detected increased Th1 cells, IL-17A^+^ Th17 cells, and Tregs (Figure 1, H and I). Additionally, the frequency of IFN-γ^+^CD8^+^ T cells was increased in liver but not eWAT of HFD-fed mice (Figure 1I). In line with unaltered expression of activation markers on splenic DCs, T cell cytokine expression in the spleen was largely unaffected in obese mice (Supplemental Figure 2B). These data suggest that the changing microenvironment in metabolic tissues during obesity alters DC activation and, consequently, DC-mediated T cell responses.

As a bioenergetic sensor, LKB1 was recently shown to be a critical regulator of DC biology and T cell responses in vivo (20–22). We next investigated LKB1 signaling in spleen, eWAT, and liver DCs by flow cytometry to determine its potential role in tissue-specific DC responses to HFD. Interestingly, we found a marked increase in phosphorylation of Ser431-LKB1 specifically in hepatic DCs from obese mice, suggesting that signaling to LKB1 within DCs is altered during high-fat feeding, whereas Ser79-ACC phosphorylation — as a proxy for activity of AMPK, the canonical downstream target of LKB1 — was unchanged (Figure 1, J and K, and Supplemental Figure 2C). Together, these findings indicate that obesity-induced changes in the hepatic microenvironment may affect signaling to LKB1 in DCs, and this signaling is associated with altered hepatic T cell responses.

### LKB1 deficiency in CD11c-expressing cells aggravates obesity-induced metabolic dysfunctions.

**Figure 2:**
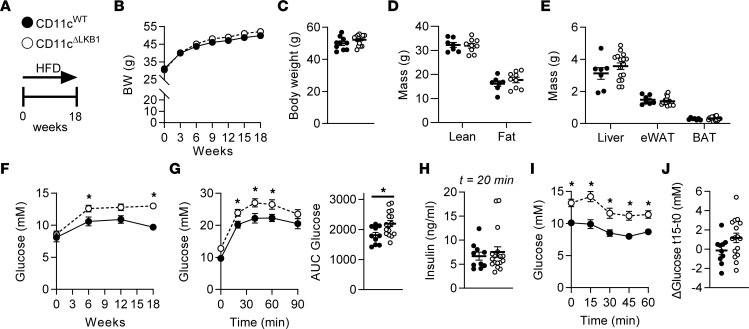
Deletion of LKB1 in DCs aggravates whole-body glucose intolerance and insulin resistance in obese mice. (**A**) CD11c^WT^ (black symbols) and CD11c^ΔLKB1^ (open symbols) mice were fed a HFD for 18 weeks. (**B** and **C**) Body weight was monitored throughout the experiment. (**D** and **E**) Body composition (**D**) and weights of liver, eWAT, and BAT (**E**) were measured at the end of the experiment. (**F**) Fasting blood glucose was measured at the indicated weeks. (**G**) An i.p. glucose tolerance test (GTT) was performed 1 week before sacrifice. Blood glucose levels were measured at the indicated time points, and the AUC of the glucose excursion curve was calculated. (**H**) Plasma insulin was measured at 20 minutes after glucose injection during i.p. GTT. (**I**) An i.p. insulin tolerance test was performed 1 week before sacrifice. Blood glucose levels were measured at the indicated time points. (**J**) Delta glucose values between 0 and 15 minutes. Data shown are a pool of 2 independent experiments. Data are expressed as mean ± SEM. Statistical analyses were performed using unpaired *t* test (**C**–**E**, **G**, **H**, and **J**) or 2-way ANOVA followed by Fisher’s post hoc tests (**B**, **F**, **G**, and **I**). *P<0.05 versus CD11c^WT^ (*n* = 7–17 mice per group).

To study the role of LKB1 in DCs in the context of obesity-induced metabolic inflammation, we crossed *Stk11^fl/fl^* mice to *Itgax^Cre^* mice to generate mice with CD11c-specific deletion of LKB1. We and others have previously shown that LKB1 RNA and protein is efficiently deleted from CD11c-expressing DCs in this model and only partially from splenic macrophages (21, 22). Male conditional KO (CD11c^ΔLKB1^) and Cre^–^ littermate control (CD11c^WT^) mice were fed a HFD for 18 weeks (Figure 2A), and it did not result in differences in body weight gain or body composition between genotypes (Figure 2, B–E). Food intake, energy expenditure (EE), and carbohydrate (CHO) and FA oxidation were also not affected by loss of LKB1 in CD11c-expressing cells (Supplemental Figure 3). However, despite similar levels at baseline, CD11c^ΔLKB1^ mice developed higher fasting blood glucose levels than CD11c^WT^ littermates after 6 weeks on HFD, and this trend was sustained throughout the experiment (Figure 2F). Furthermore, the glucose excursion during the glucose tolerance test (GTT) was larger in CD11c^ΔLKB1^ than in CD11c^WT^ mice (Figure 2G), while glucose-induced insulin levels were similar (Figure 2H), suggesting a stronger impairment in HFD-induced insulin resistance. Although significant differences in glucose levels were already present at baseline, complexifying the interpretation of the data, similar results were obtained during an insulin tolerance test (ITT) (Figure 2I), with a borderline significant (*P* = 0.08) lower glucose drop in the acute response to insulin bolus in CD11c^ΔLKB1^ compared with CD11c^WT^ mice (Figure 2J). Altogether, this suggests that LKB1 expression in CD11c^+^ cells is important for restraining metabolic dysfunctions in mice during HFD-induced obesity.

### Deletion of LKB1 in DCs promotes hepatic Tregs and Th17 cells and exacerbates hepatic steatosis.

We next determined if the exacerbated metabolic dysfunctions observed in obese CD11c^ΔLKB1^ mice could be driven by tissue-specific immunometabolic changes**.** In eWAT, total leukocyte count and relative abundances of eosinophils, neutrophils, monocytes, and macrophages were unaffected**.** However, expression of the obesity-associated macrophage marker CD11c was reduced in CD11c^ΔLKB1^, while the absolute number of CD11c^+^ macrophages per gram eWAT and CD86 expression were unchanged (Supplemental Figure 4, A–F)**.** In line with our previous findings that LKB1-deficient DCs are more migratory (22), we found that the relative abundance and numbers of DCs per gram eWAT were decreased in obese CD11c^ΔLKB1^ mice while frequencies of cDC subsets between genotypes remained similar (Supplemental Figure 4, G and H)**.** Since previous work revealed that LKB1-deficient DCs induced Tregs and effector Th17 cells mostly in lymphoid tissues of lean mice (21, 22), we next assessed whether these Th subsets are affected in eWAT from obese CD11c^ΔLKB1^ mice**.** Despite similar CD4^+^ T cell abundance, frequencies of FOXP3^+^ Tregs and IL-17A^+^ Th17 cells within the CD4^+^ T cell pool were increased in eWAT from obese CD11c^ΔLKB1^ mice, while IL-5^+^ Th2 cells were not (Supplemental Figure 4, I–K)**.** However, when expressed as a number of cells per gram eWAT, neither Tregs nor Th17 cells were significantly increased (Supplemental Figure 4L)**.** Furthermore, adipocyte mean diameter and size distribution were not affected in obese CD11c^ΔLKB1^ mice (Supplemental Figure 4, M–O)**.**

**Figure 3:**
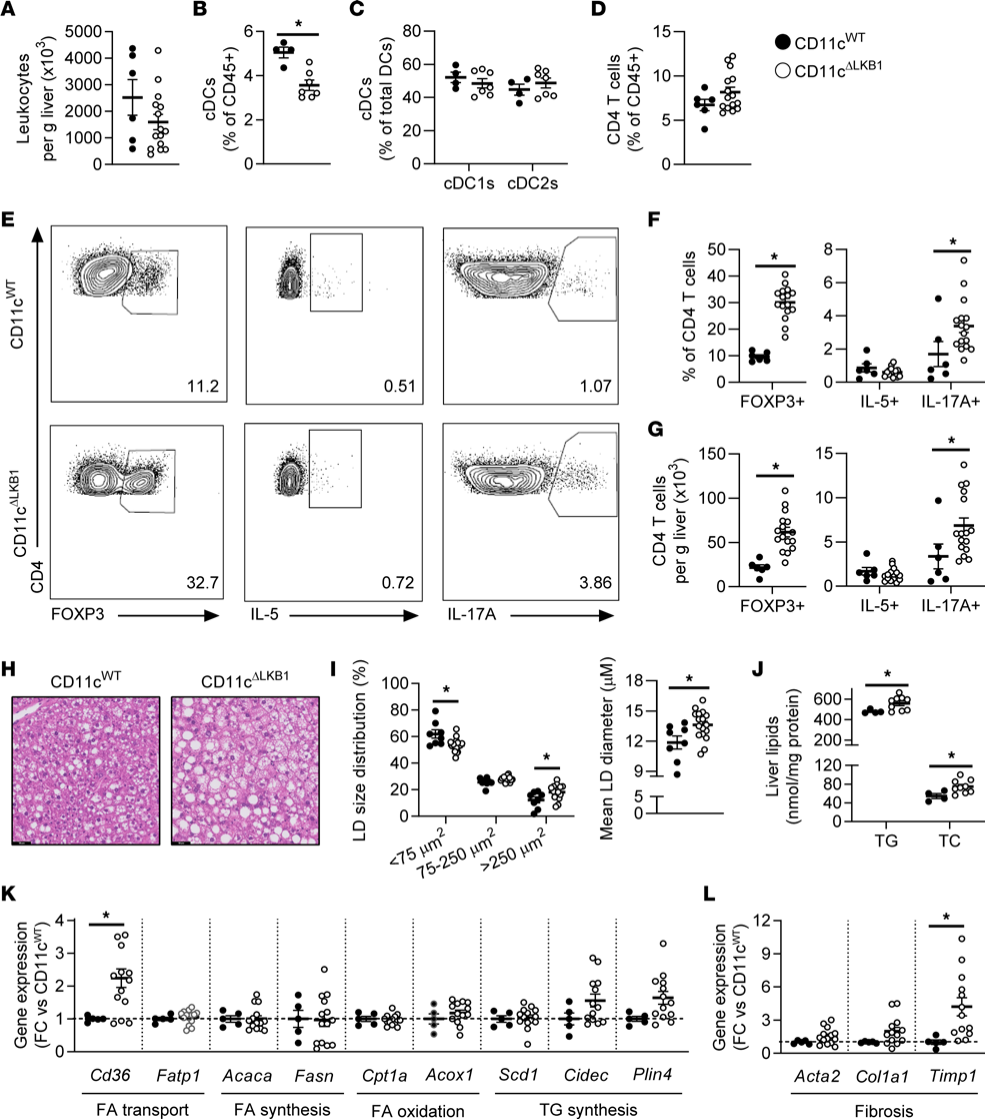
Obese CD11c^ΔLKB1^ mice are more susceptible to HFD-induced hepatic steatosis and have increased hepatic Treg and Th17 cells. CD11c^WT^ (black symbols) and CD11c^ΔLKB1^ (open symbols) mice were fed a HFD for 18 weeks**.** (**A**–**D**) At sacrifice, liver was collected and immune cells were isolated**.** Total leukocytes per gram liver were quantified (**A**). Percentages of DCs (**B**), cDC subsets (**C**), and CD4^+^ T cells (**D**) were determined by flow cytometry**.** (**E**–**G**) Liver leukocytes were restimulated with PMA and ionomycin in the presence of Brefeldin A for intracellular cytokine detection**.** Representative plots (**E**) and percentages of FOXP3^+^ Tregs, IL-5^+^ Th2, and IL-17A^+^ Th17 cells were determined as frequencies of CD4^+^ T cells (**F**) or cells per gram liver (**G**)**.** (**H**) A part of liver was sectioned and H&E stained**.** (**I**) Lipid droplet size distribution and mean lipid droplet diameter were quantified from H&E-stained slides**.** (**J**) Hepatic triglyceride (TG) and total cholesterol (TC) contents were determined**.** (**K** and **L**) Hepatic gene expression of genes involved in lipid metabolism (**K**) and fibrosis (**L**) was measured by qPCR**.** Data shown are a pool of 2 independent experiments, except for **B**, **C**, and **J.** Data are expressed as mean ± SEM**.** Statistical analyses were performed using unpaired *t* tests (**A**–**L**) or 2-way ANOVA followed by Fisher’s post hoc tests (**I**)**.** *P < 0.05 versus CD11c^WT^ (*n* = 6–17 mice per group for **A** and **D**–**I**; *n* = 4–9 mice per group for **B**, **C**, and **J**)**.** Scale bar: 50 μm**.**

In the liver, the abundance of total leukocytes, eosinophils, neutrophils, monocytes and Kupffer cells was unchanged in obese CD11c^ΔLKB1^ mice when compared with CD11c^WT^ littermates (Figure 3A and Supplemental Figure 5). However, expression of CD11c and CD86 on Kupffer cells was either significantly or tended to be decreased, and numbers of CD11c^+^ Kupffer cells were decreased in CD11c^ΔLKB1^ mice (Supplemental Figure 5E). The frequency and number of DCs per gram liver were reduced in CD11c^ΔLKB1^ mice, although the relative abundance of DC subsets remained similar (Figure 3, B and C, and Supplemental Figure 5, F and G). Importantly, deletion of *Stk11*, the gene encoding LKB1, was complete in hepatic type 2 cDCs (cDC2s) from CD11c^ΔLKB1^ mice while displaying only a partial deletion in CD11c-expressing Kupffer cells, likely due to lower CD11c expression in these myeloid cells (Supplemental Figure 5, I and J), as previously reported (21, 22, 26). Strikingly, the proportions of liver Tregs and Th17 cells were significantly increased in mice with LKB1-deficient DCs in comparison with LKB1-sufficient controls (Figure 3, D–G). Moreover, the livers of CD11c^ΔLKB1^ obese mice exhibited enhanced hepatic steatosis characterized by larger lipid droplets (LDs) when compared with WT littermates (Figure 3, H and I). Consistent with this, triglyceride (TG) and total cholesterol (TC) levels were also higher (Figure 3J) in the livers of HFD-fed CD11c^ΔLKB1^ mice and were associated with increased expression of genes encoding the FA transporter CD36, the LD coating molecules FSP27 (*Cidec*) and Perilipin-4 (*Plin4*), and some fibrotic markers (Figure 3, K and L).

These results show that deletion of LKB1 in DCs induces a potent increase in Tregs and Th17 cells in the liver and exacerbates hepatic steatosis in obese mice.

### IL-17A neutralization prevents exacerbated obesity-induced metabolic dysfunctions in CD11c^ΔLKB1^ mice.

**Figure 4:**
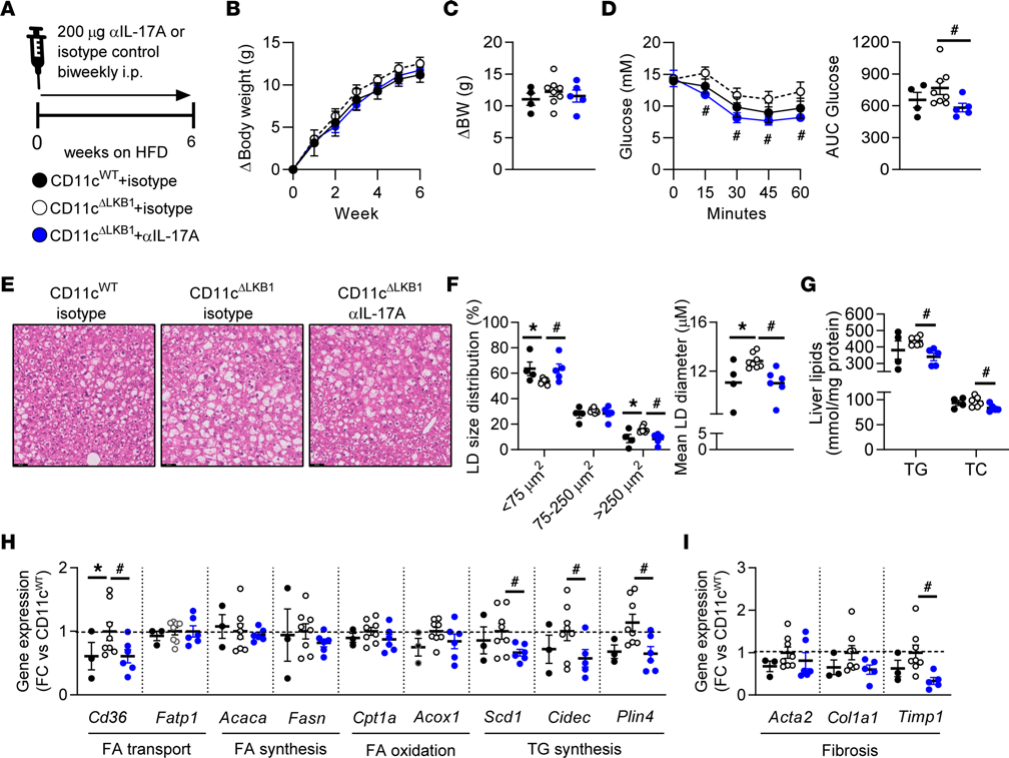
IL-17A neutralization rescued insulin resistance and hepatic steatosis in CD11c^ΔLKB1^ mice. (**A**) CD11c^WT^ (black symbols) and CD11c^ΔLKB1^ mice were fed a HFD for 6 weeks while concomitantly receiving a biweekly i.p. treatment with IL-17A neutralizing antibodies (blue symbols) or isotype control (open symbols). (**B** and **C**) Body weight gain was monitored throughout the experiment. (**D**) An i.p. insulin tolerance test was performed at week 6. (**E**) At sacrifice, a piece of liver was sectioned and H&E stained. (**F**) Lipid droplet size distribution and mean lipid droplet diameter were quantified from H&E-stained slides. (**G**) Hepatic TG and TC content were determined. (**H** and **I**) Hepatic gene expression of genes involved in lipid metabolism (**H**) and fibrosis (**I**) was measured by qPCR. Data shown are a pool of 2 independent experiments. Data are expressed as mean ± SEM. Statistical analyses were performed using 2-way ANOVA (**B**, **D**, and **F**) or 1-way ANOVA (**C** and **D**–**I**) followed by Fisher’s post hoc tests.*P < 0.05 versus CD11c^WT^; ^#^*P* < 0.05 versus CD11c^ΔLKB1^ + isotype control (*n* = 4–8 mice per group). Scale bar: 50 μm.

WAT and liver Th17 cells have consistently been linked to obesity-induced metabolic dysfunctions (13, 27) — and hepatic steatosis, in particular (12, 14–16). Accordingly, we observed elevated IL-17A–expressing CD4^+^ T cells in the livers of obese mice lacking LKB1 in DCs, a feature that was associated with enhanced hepatic steatosis. Therefore, to investigate the contribution of increased Th17 cells to worsened metabolic dysfunctions in CD11c^ΔLKB1^ obese mice, we treated them with either neutralizing antibodies for the Th17 effector cytokine IL-17A or isotype control during the first 6 weeks on HFD (Figure 4A). IL-17A neutralization did not impact body weight gain (Figure 4, B and C) or hepatic Treg and Th17 cell abundances in CD11c^ΔLKB1^ mice (Supplemental Figure 6). However, IL-17A blockade led to significantly improved whole-body insulin sensitivity (Figure 4D), and it reduced hepatic steatosis and LD diameter to levels comparable with CD11c^WT^ littermates (Figure 4, E–G). Interestingly, the increased hepatic gene expression of *Cd36*, LD, and fibrotic markers in HFD-fed CD11c^ΔLKB1^ mice was also rescued following IL-17A neutralization (Figure 4, H and I). Thus, increased IL-17A in CD11c^ΔLKB1^ mice plays a central role in promoting liver steatosis and metabolic dysfunction during HFD-induced obesity. Our findings suggest that LKB1 in DCs mitigates hepatic inflammation during the development of obesity by restraining Th17 responses.

**Figure 5:**
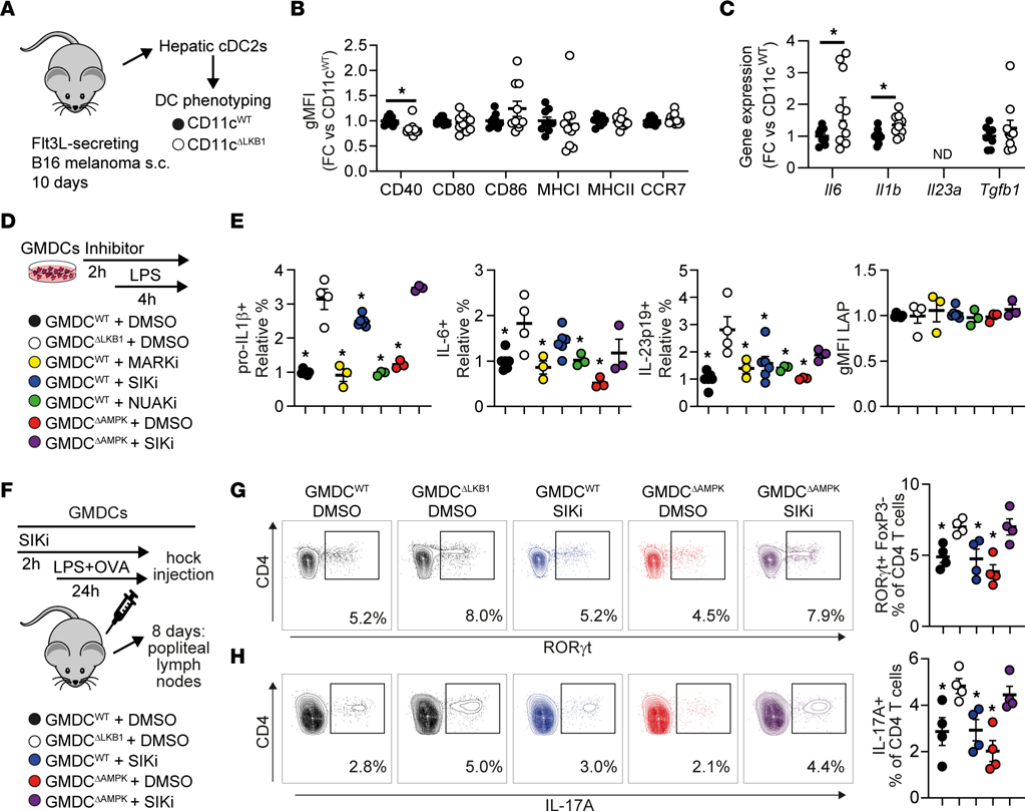
LKB1 deficiency in DCs increases a Th17 response, which is mediated via its downstream targets AMPK and SIK. (**A**–**C**) CD11c^WT^ (black symbols) and CD11c^ΔLKB1^ (open symbols) mice were s.c. injected with Flt3L-secreting B16 melanomas to expand the DC pool. After 10 days, hepatic cDC2s were sorted by FACS for DC phenotyping (**A**). Expression of indicated DC markers was measured by flow cytometry (**B**). Expression of indicated genes was measured by qPCR after ex vivo overnight LPS stimulation (**C**). (**D** and **E**) GM-CSF cultured BM-derived DCs (GMDCs) from CD11c^WT^ (WT) mice and CD11c^ΔAMPK^ (AMPK KO) mice were first treated with inhibitors targeting LKB1 downstream targets or DMSO for 2 hours before LPS stimulation in the presence of Brefeldin A for 4 additional hours, and compared with CD11c^ΔLKB1^ GMDCs (LKB1 KO). Pro–IL-1β–, IL-6–, IL-23p19–, and LAP-expressing GMDCs were quantified by intracellular cytokine staining/flow cytometry and normalized to WT-DMSO. (**F**–**H**) GMDCs (CD11c^WT^, CD11c^ΔLKB1^, or CD11c^ΔAMPKα1^) were treated with DMSO or SIK-inhibitor for 2 hours and stimulated with OVA and LPS for 24 hours, before being injected into the hock of WT mice. After 8 days, draining popliteal lymph nodes were harvested and RORγt^+^ and IL-17A^+^ Th17 cells were evaluated by flow cytometry. Data are expressed as mean ± SEM. Statistical analyses were performed using unpaired *t* tests (**B** and **C**) or 1-way ANOVA followed by Dunnett’s post hoc tests (**E**–**H**). *P < 0.05 versus LKB1 KO (*n* = 9–10 mice per group for **A**–**C**; *n* = 3–6 biological replicates per group for **E** and *n* = 4 biological replicates per group for **G** and **H**).

To explore a direct role for LKB1-deficient DCs in promoting Th17 responses, we sorted hepatic cDC2s, the main CD4^+^ T cell–priming subset shown to induce Th17 priming (28), from lean CD11c^WT^ and CD11c^ΔLKB1^ mice that were s.c. injected with Flt3L-secreting B16 melanomas to expand the in vivo DC pool (Figure 5A). Surface expression of activation markers on hepatic cDC2s was largely unchanged during homeostasis (Figure 5B). LPS-induced expression of the Th17-polarizing cytokines *Il6* and *Il1b* was enhanced in LKB1-deficient hepatic cDC2s when compared with controls, whereas *Il23a* was undetectable and *Tgfb1* was unchanged (Figure 5C). These results show that LKB1 deficiency in DCs promotes production of cytokines known to favor Th17 polarization, suggesting that LKB1 in DCs restrains Th17 polarization by limiting IL-1β and IL-6 production.

### The LKB1 downstream targets SIK and AMPK in DCs regulate Th17 responses.

Having demonstrated that LKB1 loss in DCs exacerbates obesity-induced metabolic dysfunctions in an IL-17A–dependent fashion, we next investigated the signaling mediators downstream of LKB1 involved in the Th17 priming function of DCs. The LKB1-AMPK axis represents a central node in the regulation of cellular energetics, where LKB1 promotes the downstream activation of AMPK though direct phosphorylation of its catalytic α-subunit (25). To assess whether AMPK is involved in the impaired metabolic homeostasis observed in CD11c^ΔLKB1^ obese mice, we generated CD11c^ΔAMPKα1^ mice, in which AMPKα1, the main α-subunit expressed by DCs (29), is deleted in these cells (Supplemental Figure 7A) (22). We next fed them and their CD11c^WT^ littermates an HFD for 18 weeks (Supplemental Figure 7B). Surprisingly, none of the abovementioned detrimental immunometabolic changes observed in CD11c^ΔLKB1^ obese mice — i.e., increased fasting glucose levels, glucose intolerance, insulin resistance, and hepatic Tregs and Th17 cells — were recapitulated in HFD-fed CD11c^ΔAMPKα1^ mice (Supplemental Figure 7). These data indicate that increased hepatic Th17 responses seen in CD11c^ΔLKB1^ mice are not strictly dependent on AMPK.

In addition to AMPK, LKB1 phosphorylates several other downstream AMPK-related kinases including MARK1-4, SIK1-3, NUAK1-2, BRSK1-2, and SNRK (23, 24). We therefore investigated which LKB1 targets may contribute to altering DC function by analyzing published data sets for their expression in hepatic cDCs, as well as mature GM-CSF–elicited BM DCs (GMDCs) (30–32). The expression profiles were almost identical between primary hepatic cDCs and GMDCs, showing that all these kinases were expressed to a significant level, with the notable exception of *Mark1*, *Brsk2*, and *Prkaa2* (encoding AMPKα2), confirming that only the catalytic AMPKα1 isoform is expressed by DCs (29). *Brsk1* and *Nuak1* were solely expressed in hepatic cDCs or GMDCs, respectively (Supplemental Figure 8, A and B). We next determined their role in driving Th17 responses by DCs. To this end, CD11c^WT^ GMDCs were treated with inhibitors of SIK, MARK, and NUAK families prior to LPS stimulation, and intracellular levels of Th17-polarizing cytokines were assessed by flow cytometry and compared with CD11c^ΔLKB1^ and CD11c^ΔAMPKα1^ GMDCs (Figure 5D and Supplemental Figure 8, C and D). Largely consistent with liver-derived cDC2s from CD11c^ΔLKB1^ mice, LKB1-deficient GMDCs displayed upregulated LPS-induced expression of pro–IL-1β, IL-6, and IL-23p19 when compared with WT GMDCs, whereas latency-associated peptide (LAP) expression, as a proxy for TGF-β production, was unchanged (Figure 5E). Strikingly, inhibition of SIKs, but none of the other LKB1 downstream kinases, recapitulated the cytokine profile of LKB1-deficient GMDCs (Figure 5E), identifying SIKs in DCs as potential regulators of Th17 responses.

To directly demonstrate a role for DC-intrinsic SIK in DC-mediated Th17 priming in vivo, we next performed adoptive transfer of LPS- and ovalbumin-pulsed GMDCs into WT recipient mice and assessed T cell cytokine profiles in the draining popliteal lymph nodes after 8 days (Figure 5F). Transfer of LKB1-deficient GMDCs resulted in an increased Th17 response, as indicated by elevated expression of RORγt, the canonical Th17 transcription factor, and IL-17A when compared with both CD11c^WT^ and CD11c^ΔAMPKα1^ GMDCs (Figure 5, G and H, and Supplemental Figure 8F). However, adoptive transfer of WT GMDCs after SIK inhibition alone did not increase the Th17 response (Figure 5, G and H, and Supplemental Figure 8F). This led us to hypothesize that a combination of LKB1 downstream targets, as previously reported in other contexts (33, 34), may be engaged to restrict Th17 responses. Indeed, when SIK inhibition was performed on AMPK-deficient GMDCs, these cells fully recapitulated the cytokine profile and the Th17-polarizing capacity of LKB1-deficient GMDCs (Figure 5, E–H, and Supplemental Figure 8F), suggesting that LKB1 in DCs constrains Th17 responses via a dual regulatory mechanism involving both AMPK and SIK.

Collectively, our data indicate that LKB1 signaling in DCs controls hepatic Th17 differentiation and metabolic homeostasis in obese mice, and we propose a dual role exerted by AMPK and SIK downstream of LKB1 in repressing Th17 responses.

## Discussion

The bioenergetic sensor LKB1 was recently shown to be a critical regulator of DC metabolism, activation, and T cell priming functions (20–22). Whether LKB1 signaling in DCs links the changing immunometabolic microenvironment during obesity with altered DC function and, ultimately, whole-body metabolic dysfunctions remained unclear. Here, we report that obesity increased LKB1 phosphorylation in hepatic DCs. Deletion of LKB1 from DCs increased hepatic Th17 cells and aggravated HFD-induced metabolic dysfunctions in obese mice. These immunometabolic defects were restored by neutralizing the Th17 effector cytokine IL-17A. LKB1-deficient DCs displayed a potentiated capacity to promote a Th17 response that was recapitulated by pharmacological/genetic inhibition of both AMPK and SIK in DCs, uncovering a role for an LKB1-AMPK/SIK axis in restraining DC-mediated pathogenic Th17 cell differentiation and thereby controlling whole-body metabolic homeostasis.

Although DCs accumulate in WAT and liver during obesity and contribute to whole-body insulin resistance (5–8), the underlying mechanisms are incompletely understood. Indeed, obese *Flt3l*^–/–^ mice lacking DCs and *Ccr7*^–/–^ mice with impaired DC migration displayed reduced metabolic inflammation and insulin resistance, suggesting they have a central role in the development of metabolic dysfunctions (6, 7). Here, we report that DCs from eWAT and liver, but not spleen, of obese mice display increased expression of activation markers, indicating that the obesity-induced changes in the metabolic tissue microenvironment enhance DC activation. Interestingly, both eWAT and liver DCs from obese mice expressed higher levels of CCR7, suggestive of increased migration to draining lymph nodes, where they can prime inflammatory T cells. Consistent with an increased proinflammatory activation profile of the DCs, we found that obesity altered the CD4^+^ Th cell pool in eWAT and liver, but not spleen, favoring Th1 cells at the expense of Th2 cells and Tregs in eWAT and increasing Th1 cells, Th17 cells, and Tregs in the liver. Some of these obesity-induced changes in Th subsets in metabolic tissues have been reported previously (11, 16). Moreover, XCR1^+^ cDC1s, efficient at cross-presenting antigens to CD8^+^ T cells, were reported to increase hepatic steatosis and contribute to liver pathology, which was associated with inflammatory T cell reprogramming in the liver-draining lymph nodes (8). Congruent with this, we found increased HFD-induced IFN-γ^+^CD8^+^ T cells in the liver, most likely resulting from increased cDC1-mediated priming.

In addition to increased DC activation and altered T cell priming in metabolic tissues, we observed a significant increase of Ser431-LKB1 phosphorylation in hepatic DCs of obese mice. LKB1 is phosphorylated at Ser431 by protein kinase C ζ (PKCζ) (35), p90 ribosomal S6 kinase (p90-RSK), and cAMP-dependent protein kinase A (PKA) (36). Although the exact consequence of phosphorylation at this site remains unclear, the residue corresponding to murine Ser431 is conserved in all organisms, suggesting that its phosphorylation may play a role in modulating LKB1 signaling. Despite a lack of a phenotype and normal AMPK activation in knock-in mice carrying a homozygous Ser431 to alanine mutation in LKB1 (37), it has been suggested that Ser431 phosphorylation could promote nuclear export of LKB1 and phosphorylation of some of its cytoplasmic substrates (35).

Interestingly, Ser431-LKB1 phosphorylation was unchanged in splenic and eWAT DCs, indicating that obesity-induced changes in the hepatic microenvironment may specifically alter LKB1 signaling in liver-associated DCs and change its effector functions. Obesity induces persistent changes in the gut microbiota and induces endotoxemia through increased gut permeability (38). As a result, the gut and serum metabolome is altered (39, 40), promoting NAFLD pathogenesis through a gut-liver axis (41). Since LPS injection has been reported to acutely increase Ser431-LKB1 phosphorylation in whole lung and liver lysates; in immortalized Raw264.7 macrophages (42), one may hypothesize that obesity-induced endotoxemia might contribute to increased pLKB1 levels in hepatic DCs from obese mice. Of note, in Raw264.7 macrophages, LPS-induced Ser431-LKB1 phosphorylation suppressed NF-κB signaling, suggesting that increased pLKB1 in hepatic DCs from obese mice may serve as a feedback mechanism to keep inflammation in check. In addition, butyrate, a short-chain FA produced by commensal bacteria that metabolize indigestible fiber, was recently shown to increase Ser431-LKB1 phosphorylation in HepG2 hepatocytes (43), providing conceptual evidence that gut metabolites may alter LKB1 phosphorylation in liver-resident cells. However, how LKB1 phosphorylation and other posttranslational modifications such as farnesylation (37) affect LKB1 signaling and how such modifications may be selectively modulated in hepatic DCs during obesity, remain interesting future areas of research.

Deletion of LKB1 from DCs increased hepatic Tregs and Th17 cells in obese mice. The role of Tregs in the regulation of metabolic homeostasis has become controversial over recent years. Hepatic Tregs were reported to control hepatic inflammation and inhibit NASH development (44), while adipose tissue Tregs have been shown to both protect against (45, 46) and aggravate (47–49) metabolic dysfunctions. However, we clearly demonstrate that neutralizing the Th17 effector cytokine IL-17A rescued the metabolic perturbations in HFD-fed CD11c^ΔLKB1^ mice, indicating an important role for increased Th17 cells rather than Tregs in explaining the metabolic phenotype of obese CD11c^ΔLKB1^ mice.

Obesity-induced hepatic Th17 cells and IL-17A signaling have consistently been reported to impair insulin sensitivity and promote hepatic steatosis and fibrosis in both HFD-fed mice and patients with NAFLD/NASH (12–16, 50–52). Th17 differentiation is dependent on the cytokines IL-6, TGF-β, IL-1β, and IL-23 (53, 54), but different combinations can lead to different degrees of pathogenicity. In the context of experimental autoimmune encephalitis, IL-6– and TGF-β–induced Th17 cells were not pathogenic, whereas IL-6–, IL-1β–, and IL-23–induced Th17 cells were pathogenic (55, 56). Furthermore, development of Th17 cells in vivo is dependent on SIRPα/CD172a expression on DCs (28, 57), a marker of cDC2s that efficiently prime CD4^+^ T cells. We previously showed that LKB1-deficient splenic cDC2s produced higher levels of IL-6 (22). In addition, others showed that mRNA expression of *Il6*, *Tgfb2*, and *Il23a* tended to be increased in LKB1-deficient total splenic DCs compared with WT DCs, whereas expression of *Tgfb1* was decreased and *Tgfb3* was similar (21). Moreover, increased Th17 priming by LKB1-deficient splenic DCs was at least partly dependent on IL-6 — but not TGF-β (21). Here, we found that LPS-stimulated LKB1-deficient GMDCs expressed significantly enhanced levels of IL-6, pro–IL-1β, and IL-23p19, while TGF-β production was not affected. In addition, LKB1-deficient hepatic cDC2s expressed increased levels of *Il6* and *Il1b*, whereas *Tgfb1* was unchanged and *Il23a* undetectable. These data indicate that LKB1-deficient hepatic cDC2s displayed a cytokine profile that favors the development of pathogenic Th17 cells. Recent single-cell transcriptomics analysis of hepatic Th17 cells from HFD-fed obese mice identified 2 subsets, of which 1 was enriched during obesity. The accumulation of this inflammatory hepatic Th17 (ihTh17) subset was regulated through a CXCL9/10-CXCR3 axis, and these cells were sufficient to exacerbate NAFLD pathogenesis through glycolysis-facilitated production of proinflammatory cytokines IL-17A, TNF, and IFN-γ (16). Moreover, increased IL-6, TGF-β, IL-1β, and IL-23 levels in steatotic livers were also reported in this study, suggesting involvement of DCs in generating these ihTh17 cells. Given their role in promoting NAFLD pathogenesis, it is tempting to speculate that LKB1-deficient hepatic DCs promote accrual of these ihTh17 cells.

Upon binding to IL-17RA, IL-17 promotes the polyubiquitination of the TNF receptor associated factor 6 (TRAF6) adaptor protein and downstream activation of CCAAT/enhancer-binding protein α (C/EBPα), MAPKs, and NF-κB pathways (58). Interestingly, TRAF6 ubiquitination has been shown to be increased in HFD-fed mice and to be associated with fatty liver (59), while its inhibition was recently shown to alleviate hepatic steatosis and liver fibrosis (60). Interestingly, C/EBPα is involved in the transcriptional regulation of CD36 through direct binding to the *Cd36* gene promoter (61). CD36 is a multifunctional cell-surface scavenger receptor widely expressed in the liver that, through facilitating FA uptake (62), has been shown to be positively associated with hepatic TG content in both obese mice and humans (63–65). CD36 indeed plays a central role in the development of NAFLD, notably by promoting hepatic de novo lipogenesis (66), and its hepatocyte-specific deletion attenuates fatty liver in HFD-fed mice (67). It is tempting to speculate that the elevated hepatic Th17 response of HFD-fed CD11c^ΔLKB1^ mice contributes to worsened hepatic steatosis and insulin resistance through TRAF6-C/EBPα–mediated enhanced *Cd36* expression. Further studies are required to elucidate the exact molecular mechanisms linking hepatic Th17 cells to LDs and metabolic dysfunctions.

Deletion of AMPKα1 in DCs did not recapitulate the immunometabolic phenotype of CD11c^ΔLKB1^ obese mice. Indeed, we and others have recently shown that LKB1 functions independently of AMPK in governing Tregs and Th17 cell differentiation (21, 22), which corresponds with a growing line of research showing AMPK-independent effects of LKB1 in immune cells (33, 42). However, we here show that AMPK in DCs regulates Th17 responses in vivo in combination with SIKs. SIK inhibition increased expression of the Th17-polarizing cytokines IL-6 and pro–IL-1β, while AMPK deficiency alone had no effects on Th17-polarizing cytokines in GMDCs. Interestingly, one report suggests that AMPK activation may suppress IL-23 in human monocyte–derived DCs (68). Largely congruent with these data, we found that AMPK-deficiency may boost IL-23 and IL-1β production in SIK-inhibited DCs. Apart from potentiation effects of loss of AMPK signaling on Th17 polarizing cytokine production, LKB1-AMPK signaling has also been shown to restrain Th17 responses by limiting DC–T cell synapse formation (69). Alternatively, AMPK has been described to promote catabolic metabolism in cells, including DCs (18, 70), while glycolysis and mTOR-mediated anabolic metabolism were shown to play a role in Th17 polarization by DCs (17). Although the metabolic requirements for Th17 polarization by DCs are likely context dependent, it is tempting to speculate that AMPK deletion may provide the metabolic requirements of DCs for promoting Th17 polarization, while SIK inhibition provides the required cytokine profile. However, this warrants further study.

The SIK family consists of 3 isoforms, SIK1–3, and is involved in regulating hepatic gluconeogenesis, lipid metabolism, and tumorigenesis (71), although its underlying mechanisms are only beginning to be understood. SIKs control the phosphorylation and nucleocytoplasmic transport of class IIa histone deacetylases (HDACs) and cAMP-regulated transcriptional coactivators (CRTCs), identifying a role for SIKs in transcriptional regulation (72). CRTC is a coactivator of cAMP response element-binding protein (CREB) (73), and the promotors of *Il6*, *Il1b*, and *Il23a* all contain CREB binding sites (74–76). One could, thus, speculate that inhibition of SIKs may promote CRTC nuclear transport, thereby promoting transcription of Th17-polarizing cytokines. In support of this, SIK1 and SIK3 were shown to control IL-6 production in tumor cells (77) and IL-6 and IL-1β production in Raw264.7 macrophages (78). Conversely, pharmacological inhibition of SIKs was also reported to suppress proinflammatory cytokine production in DCs and macrophages (79, 80). Since SIK family members display functional redundancy in some settings (72), future studies are required to identify which SIK family members control expression of Th17-polarizing cytokines and what the mechanistic underpinnings are.

Although our data strongly argue in favor of a DC-intrinsic mechanism, we cannot formally rule out that deletion of LKB1 in other CD11c-expressing cells, such as macrophages, contribute to the immunometabolic phenotype. Indeed, LysM^Cre^-driven LKB1 deletion in macrophages has been shown to increase LPS-induced proinflammatory cytokine production (42). However, these LysM^ΔLKB1^ mice have unchanged Treg numbers (20), indicating that LKB1 deletion from macrophages does not alter T cell responses in vivo. In addition, congruent with previous work, only partial KO of *Stk11* was observed in macrophages from CD11c^ΔLKB1^ mice (21, 26), likely attributable to lower CD11c expression by macrophages as compared with DCs. CD11c on macrophages is considered a proinflammatory/metabolic inflammation–associated marker in eWAT and liver of HFD-fed mice. We demonstrated that CD11c expression was downregulated on eWAT and liver macrophages of CD11c^ΔLKB1^ mice, suggesting that fewer proinflammatory macrophages are present and that efficiency of Cre-mediated recombination is further hampered. Furthermore, we found that expression of the proinflammatory marker CD86 on both eWAT and liver macrophages was unchanged, as was ex vivo LPS-induced TNF and IL-6 production by CD11c^+^ macrophages from CD11c^ΔLKB1^ mice (data not shown). This makes it unlikely that macrophages play a dominant role in the immunometabolic phenotype of CD11c^ΔLKB1^ mice. However, the use of *Zbtb46^Cre^* (zDC^Cre^) mice, a model for cDC-specific conditional gene deletion that does not affect macrophages (81), may also be considered for future study.

Altogether, our data reveal a key role for LKB1 signaling in liver-resident DCs in limiting liver-specific and whole-body metabolic dysfunction in the context of obesity, by constraining hepatic Th17 accrual. We suggest the involvement of an LKB1-AMPK/SIK axis to control Th17-responses by DCs, potentially opening interesting therapeutic options in controlling pathogenic Th17 cell development in metabolic inflammation and other hyperinflammatory disorders.

## Methods

### Animals, diet, and treatment.

*Itgax^Cre^* (CD11c; PMID: 17591855), *Stk11^fl/fl^* (LKB1; PMID: 12226664), *Prkaa1^fl/fl^* (AMPKα1; PMID: 21124450), and WT mice, all on C57BL/6J background, were purchased from The Jackson Laboratory or Envigo and crossed, housed, and bred at the LUMC. Mice were housed in a temperature-controlled room with a 12-hour light-dark cycle and ad libitum access to food and tap water under specific pathogen–free conditions. To reduce variation due to sex hormone cycles on whole-body metabolism, male mice were used for all in vivo experiments. An a priori power calculation was done. Analysis was performed blinded to the conditions.

Eight- to 16-week-old age-matched WT, *Stk11^fl/fl^* (CD11c^WT^), *Itgax^Cre^ Stk11^fl/fl^* (CD11c^ΔLKB1^), *Prkaa1^fl/fl^* (CD11c^WT^), and *Itgax^Cre^ Prkaa1^fl/fl^* (CD11c^ΔAMPKα1^) male mice were fed a HFD (45% energy derived from fat, D12451, Research Diets) for 18–24 weeks as indicated.

For IL-17A neutralization experiments, 12- to 19-week-old age-matched CD11c^WT^ and CD11c^ΔLKB1^ mice were systematically randomized over treatment groups based on body weight and fasting blood glucose levels, and they were fed a HFD for 6 weeks while concomitant biweekly treatment with 200 μg anti–mouse IL-17A (clone 17F3) or IgG1 κ isotype control (clone MOPC-21; both BioXCell). At sacrifice, spleen, visceral WAT (epidydimal WAT [eWAT]), brown adipose tissue (BAT; intrascapular), and liver were weighed and collected for further processing.

### Body composition and indirect calorimetry.

Body composition was measured by MRI using an EchoMRI (Echo Medical Systems). Indirect calorimetry was performed in groups of 7–8 mice using a Comprehensive Laboratory Animal Monitoring System (Columbus Instruments) with free access to food and tap water. Mice were individually housed at room temperature, and a standard 12-hour light/dark cycle was maintained throughout the measurements. Mice were acclimated to the cages for a period of 48 hours before the start of 4 days of measurements at 20-minute intervals. Food intake was assessed by real-time feed weight measurements. Oxygen consumption and carbon dioxide production were measured, and based on this respirometry, EE as well as CHO and FA oxidation were calculated as previously described (82).

### Isolation of leukocytes from spleen.

Spleens were collected in 500 μL RPMI 1640 + Glutamax (Invitrogen), mechanically disrupted, and digested for 20 minutes at 37°C in medium supplemented with 1 mg/mL Collagenase D (Roche) and 2000 U/mL DNase I (Sigma-Aldrich). Digested samples were filtered through 100 μm filters and subjected to erythrocyte lysis buffer (0.15M NH_4_Cl [Baker Analyzed], 1 mM KHCO_3_ [MilliporeSigma], 0.1 mM Na_2_EDTA [Thermo Fisher Scientific]) before counting using a hemocytometer.

### Isolation of stromal vascular fraction from adipose tissue.

After a 1-minute transcardial perfusion with PBS after sacrifice, eWAT samples were collected and digested as described previously (83, 84). In short, eWAT samples were minced and incubated for 1 hour at 37°C in an incubator under agitation (60 rpm) in HEPES-buffered Krebs solution, containing 0.5–1 g/L collagenase type I from *Clostridium histolyticum* (Sigma-Aldrich), 2% (w/v) dialyzed BSA (fraction V; Sigma-Aldrich), and 6 mM D-Glucose (Sigma-Aldrich). The samples were passed through a 100 μm filter (Corning Life Sciences), which was washed with PBS supplemented with 2.5 mM EDTA and 5% FCS. After allowing the adipocytes to settle for approximately 10 minutes, the infranatant, consisting of immune cells, was collected and pelleted at 350*g* for 10 minutes at room temperature. The pellet was treated with erythrocyte lysis buffer, washed with PBS/EDTA/FCS, and counted using a hemocytometer.

### Isolation of leukocytes from liver.

Livers were collected and digested as described previously (83, 84). In short, livers were minced and incubated for 45 minutes at 37°C in RPMI 1640 + Glutamax containing 1 mg/mL collagenase type IV from *C. histolyticum*, 200 U/mL DNase (both from Sigma-Aldrich), and 1 mM CaCl_2_. The digested tissues were passed through a 100 μm cell strainer (Corning Life Sciences), which was subsequently washed with PBS/EDTA/FCS. After centrifugation (530*g*, 10 minutes at 4°C), cells were resuspended in 30 mL PBS/EDTA/FCS and spun down at 50*g* for 3 minutes at 4°C to pellet the hepatocytes. The supernatant was collected and treated with erythrocyte lysis buffer, and CD45^+^ leukocytes were isolated using LS columns and CD45 MicroBeads (35 μL beads per sample; Miltenyi Biotec) according to the manufacturer’s protocol. Isolated liver leukocytes were counted using a hemocytometer.

### Flow cytometry.

For assessing LKB1/ACC phosphorylation state in spleen, eWAT, and liver, tissues were collected and immediately minced in 1.85% formaldehyde solution (Sigma-Aldrich) and digested as described above. For confirmation of AMPK deficiency in CD11c^ΔAMPKα1^ mice, digested splenocytes were incubated for 1 hour at 37°C, before fixation in 1.85% formaldehyde solution. Isolated cell suspensions were permanently permeabilized using 100% methanol for 10 minutes at –20°C. For other purposes, cells were stained using a Fixable Aqua Dead Cell Stain Kit (Invitrogen) or Zombie UV Fixable Viability Kit (BioLegend) for 20 minutes at room temperature. Unless sorted or measured alive, cells were fixed for 1 hour at 4°C using a FOXP3/Transcription Factor Staining Buffer Set (Invitrogen, for FOXP3 and RORγT detection) or 15 minutes at room temperature using a 1.85% formaldehyde solution in PBS (Sigma-Aldrich). For detection of intracellular cytokines, isolated cells were cultured for 4 hours in RPMI 1640 + Glutamax in the presence of 10 μg/mL Brefeldin A (Sigma-Aldrich) and stimulated with either 100 ng/mL phorbol myristate acetate (PMA) and 1 μg/mL ionomycin (both Sigma-Aldrich; T cells) or 100 ng/mL LPS (Ultrapure, Invivogen; GMDCs). After 4 hours, cells were washed with PBS, stained with live/dead marker, and fixed as described above. Cell suspensions were first preincubated with 2.4G2 antibody (provided by Louis Boon, Bioceros, Utrecht, Netherlands) for blocking Fc receptors and next stained for surface markers in PBS supplemented with 0.5% BSA (Roche) and 2 mM EDTA (Sigma-Aldrich) and antibodies for 30 minutes at 4°C. For detection of phosphorylated proteins, transcription factors and intracellular cytokines, cell suspensions were stained in permeabilization buffer (eBioscience) instead. Phosphorylated Ser79-ACC and Ser431-LKB1 were stained using unconjugated rabbit anti-mouse antibodies prior to staining with other antibodies and goat anti–rabbit Alexa Fluor 647. Antibody information is provided in Supplemental Table 1, and gating strategies for tissues shown in Supplemental Figure 1. Cells were measured on a FACSCanto II, LSR II, or a Cytek Aurora 3-laser spectral flow cytometer and analyzed using FlowJo (Version 10.6, Tree Star Inc.).

### Plasma analysis.

Blood samples were collected from the tail tip of 4-hour–fasted mice using paraoxon-coated glass capillaries. Fasting blood glucose level was determined using a hand-held Glucometer (Accu-Check; Roche Diagnostics), and plasma insulin level was measured using a commercial kit as per manufacturer’s instructions (Chrystal Chem).

### Insulin and glucose tolerance tests.

Whole-body i.p. ITT and i.p. GTT were performed 1 week before sacrifice, as previously described (83, 84). In short, a bolus of insulin (0.75 U/kg body mass, NOVORAPID, Novo Nordisk) was administered i.p. to 4-hour–fasted mice, after which blood glucose levels were measured at t = 0, 15, 30, 45, and 60 minutes after insulin administration using a Glucometer. For i.p. GTT, 6-hour–fasted mice were injected i.p. with 2 g/kg total body mass of D-Glucose (Sigma-Aldrich), and blood glucose was measured at t = 0, 20, 40, 60, and 90 minutes after glucose injection using a Glucometer.

### Histological analysis.

Pieces of eWAT and liver (~30 mg) were fixed in 4% formaldehyde solution (Sigma-Aldrich), paraffin-embedded, sectioned at 4 μm, and stained with H&E. Six fields at 20***×*** magnification (total area 1.68 mm^2^) were used for the analysis of adipocyte size, crown-like structures or hepatic steatosis.

### Hepatic lipid composition.

Liver lipids were extracted as previously described (85). Liver TG and TC concentrations were measured using commercial kits (all from Instruchemie) and expressed as nanomoles per milligram of total protein content using the Bradford protein assay kit (Sigma-Aldrich).

### In vivo DC expansion, isolation, and sorting.

To expand the DC pool in vivo, 2 × 10^6^ Flt3L-secreting B16 melanoma cells (gift from Edward Pearce, Johns Hopkins University, Baltimore, Maryland, USA) in 100 μL HBSS were injected s.c. into the flank of mice. After 10 days, spleen, liver, and eWAT were harvested, digested, and processed as described earlier. cDC2s were further enriched from single-cell suspensions by positive isolation with CD11c Microbeads (Miltenyi Biotec, per manufacturer’s instructions) and FACS (MHCII^+^CD11c^+^CD64^–^F4/80^–^CD172a^+^XCR1^–^) on a BD FACSAria using a 100 μm nozzle at 20 PSI. Subsequently, sorted cDC2s were stimulated with 100 ng/mL LPS for 16 hours for assessing cytokine expression by quantitative PCR (qPCR).

### BM-derived DC cultures.

BM-derived DCs were cultured as described previously (22). Briefly, BM cells were flushed from femurs and tibias, and 5 × 10^6^ cells were plated in tissue culture–treated petri dishes (Nunc) in 10 mL of differentiation medium, consisting of RMPI 1640 Glutamax (Thermo Fisher Scientific) supplemented with 5% FCS (Thermo Fisher Scientific), 25 nM β-mercaptoethanol (Sigma-Aldrich), 100 U/mL penicillin (Sigma-Aldrich), 100 μg/mL streptomycin (Sigma-Aldrich), and 20 ng/mL of murine GM-CSF (PeproTech). Medium was refreshed on day 4 and day 7, after which — on day 10 — nonadherent GMDCs were harvested. In total, 1 × 10^5^ GMDCs were seeded in a round-bottom 96-well plate and rested overnight. The next day, GMDCs were incubated for 2 hours at 37°C with 50 μM MARK inhibitor (MARK/Par-1 Activity Inhibitor, 39621, Calbiochem), 50 nM SIK inhibitor (HG-9-91-01, Cayman Chemical), or 1 μM NUAK inhibitor (WZ 4003, Tocris).

### GMDCs adoptive transfer.

GMDCs were pretreated for 2 hours with DMSO (MilliporeSigma) or SIK inhibitor (HG-9-91-01) and subsequently stimulated for 24 hours with 100 ng/mL LPS and 100 μg/mL OVA (InvivoGen). Cells were harvested, washed and 450,000 GMDCs, suspended in 30 μL HBSS without phenol red (Thermo Fisher Scientific), and injected into the hock of WT C57BL/6J mice. After 8 days, the draining popliteal lymph nodes were harvested and the Th17 response was analyzed by measuring RORγT expression and intracellular IL-17A by flow cytometry. The IL-17A concentration was also measured after a 48-hour OVA-specific restimulation of popliteal lymph node cells using BD cytometric bead array (CBA) flex-set kits (BD Biosciences, according manufactures instructions).

### RNA-isolation and qPCR.

RNA was extracted from snap-frozen liver samples and LPS-stimulated sorted cDC2s or GMDCs using TriPure RNA Isolation (Roche) reagent. Total RNA (200–400 ng for sorted cDC2s or GMDCs; 2 μg for liver) was reverse transcribed using the M-MLV Reverse Transcriptase kit (Thermo Fisher Scientific). qPCR runs were performed on a CFX96 Real-time C1000 thermal cycler (Bio-Rad) using the GoTaq qPCR Master Mix kit (Promega). Gene expression was normalized to the housekeeping gene *Rplp0* and expressed as fold change compared with CD11c^WT^ samples. A list of primer sequences can be found in Supplemental Table 2.

### Western blot.

GMDCs were washed in PBS, snap-frozen in liquid nitrogen, and lysed in ice-cold buffer containing: 50 mM Hepes (pH 7.6) (Sigma-Aldrich), 50 mM NaF (Sigma-Aldrich), 50 mM KCl (MilliporeSigma), 5 mM NaPPi (Sigma-Aldrich), 1 mM EDTA (Thermo Fisher Scientific), 1 mM EGTA (Sigma-Aldrich), 1 mM DTT (Promega), 5 mM β-glycerophosphate (Sigma Aldrich), 1 mM sodium vanadate (Sigma-Aldrich), 1% NP40 (Sigma-Aldrich), and protease inhibitors cocktail (Complete, Roche). Proteins were separated by SDS-PAGE and transferred to a PVDF membrane. Membranes were blocked for 1 hour at room temperature in TTBS buffer (20 mM Tris–HCl [pH 7.6; Thermo Fisher Scientific], 137 mM NaCl [MilliporeSigma], and 0.25% [v/v] Tween 20 [MilliporeSigma]) containing 5% (w/v) fat-free milk. Membranes were incubated with primary antibodies overnight at 4°C, washed in TTBS buffer, incubated with horseradish peroxidase–conjugated secondary antibodies for 2 hours at room temperature, washed again, and developed using enhanced chemiluminescence. Primary antibodies included AMPK (Cell Signaling Technology, 2532) and HSP90 (Santa Cruz Biotechnology Inc., sc7947).

### Statistics.

All data are presented as mean ± SEM. Statistical analysis was performed using GraphPad Prism version 8 for Windows (GraphPad Software) with 2-tailed unpaired *t* test or 1- or 2-way ANOVA followed by Fisher’s or Dunnett’s post hoc tests. Differences between groups were considered statistically significant at *P* < 0.05.

### Study approval.

All experiments were performed in accordance with the *Guide for the Care and Use of Laboratory Animals* (National Academies Press, 2011). Mouse experiments have received approval from the Dutch Central Authority for Scientific Procedures on Animals (CCD; animal license no. AVD116002015253).

## Author contributions

HJPVDZ, ECB, MY, BE, and BG conceptualized research; HJPVDZ, ECB, JML, FO, and BG analyzed data; HJPVDZ, ECB, JML, LRP, AZD, TAP, GAH, FO, and AOF performed research; MY, BE, and BG supervised the study; and HJPVDZ, ECB, BE, and BG wrote the manuscript. HJPVDZ and ECB contributed equally to this study and should be considered as shared first author; the author order was established based on the chronological contribution to the manuscript. The same reasoning applies to senior authorship, with BE and BG having also contributed equally to the study.

## Supplementary Material

Supplemental data

Supporting data values

## Version 1 05/04/2023

In-Press Preview

## Version 2 06/08/2023

Electronic publication

## Version 3 06/20/2023

Addition of the word “and” in the antepenultimate sentence within abstract
