# Supplementary material for: Dendritic cell–intrinsic LKB1-AMPK/SIK signaling controls metabolic homeostasis by limiting the hepatic Th17 response during obesity
Source: JCI Insight. 2023 Jun 8;8(11):e157948. doi: 10.1172/jci.insight.157948 (PMC10281441; doi:10.1172/jci.insight.157948)
Supplement: Supplemental data [file jciinsight-8-157948-s290.pdf]

Supplementary Table 1: Antibodies and reagents for flow cytometry

Target	Clone	Conjugate	Source	Identifier
B220	RA3-6B2	FITC	eBioscience	11-0452
CD3	17A2	APC-eF780	eBioscience	47-0032
CD3	17A2	BV605	Biolegend	100237
CD3	17A2	FITC	eBioscience	11-0032
CD4	GK1.5	BV650	BD Biosciences	563232
CD4	GK1.5	PE-Cy7	eBioscience	25-0041
CD4	GK1.5	PerCP-eFluor 710	eBioscience	46-0041
CD8a	53-6.7	BV711	Biolegend	100759
CD8a	53-6.7	PE	eBioscience	12-0081
CD11b	M1/70	FITC	eBioscience	11-0112
CD11b	M1/70	PE-Cy7	eBioscience	25-0112
CD11c	N418	BV421	Biolegend	117330
CD11c	HL3	FITC	BD Biosciences	553801
CD11c	HL3	Horizon V450	BD Biosciences	560521
CD11c	N418	PE-Cy7	eBioscience	25-0114
CD19	MB19-1	FITC	eBioscience	11-0191
CD40	HM40-3	FITC	eBioscience	11-0402
CD44	IM7	eFluor 450	eBioscience	48-0441
CD45	30-F11	BV785	Biolegend	103149
CD45.2	104	FITC	Biolegend	109806
CD45.2	104	eFluor 450	eBioscience	48-0454
CD64	X54-5/7.1	PE	Biolegend	139304
CD64	X54-5/7.1	PE/Dazzle 594	Biolegend	139319
CD64	X54-5/7.1	PerCP-Cy5.5	Biolegend	139308
CD80	16-10A1	APC	eBioscience	17-0801
CD86	GL-1	APC/Fire 750	Biolegend	105045
CD86	GL-1	PE	BD Biosciences	553692
CD172a	P84	PE	Biolegend	144011
CD197/CCR7	4B12	PerCP-Cy5.5	Biolegend	120116
F4/80	BM8	APC	eBioscience	17-4801
F4/80	BM8	BV711	Biolegend	123147
FOXP3	FJK-16s	APC	eBioscience	17-5773
Goat-anti-Rabbit	Polyclonal	Alexa Fluor 647	Invitrogen	A21244
GR-1	RB6-8C5	FITC	BD Biosciences	553126
IFNγ	XMG1.2	FITC	eBioscience	11-7311
IL-5	TRFK5	PE	Biolegend	504303
IL-6	MP5-20F3	APC	Biolegend	504507
IL-17A	eBio17B7	PE-Cy7	eBioscience	25-7177
IL-17A	TC11-18H10.1	PerCP-Cy5.5	Biolegend	506919
IL-23p19	fc23cpg	eFluor 660	Invitrogen	50-7023
LAP	TW7-16B4	PerCP-eF710	Invitrogen	46-9821
Ly6C	HK1.4	APC-Cy7	Biolegend	128026
MHCI/H-2Kb	AF6-88.5	Pacific Blue	Biolegend	116514
MHCII	M5/114 15.2	Alexa Fluor 700	Invitrogen	56-5321
MHCII	M5/114 15.2	APC-eFluor 780	eBioscience	47-5321
MHCII	M5/114 15.2	FITC	eBioscience	11-5321
NK1.1	PK136	FITC	eBioscience	11-5941
Phospho-ACC (Ser79)	D7D11	-	Cell Signaling	11818S
Phospho-LKB1 (Ser431)	C67A3	-	Cell Signaling	3482S
Pro-IL-1β	NJTEN3	PE	Invitrogen	12-7114
RORgT	Q31-378	PE	BD Biosciences	562607
Siglec-F	E50-2440	PE	BD Biosciences	552126
XCR1	ZET	BV650	Biolegend	148220

Other reagents	Source	Identifier
LIVE/DEAD™ Fixable Aqua Dead Cell Stain Kit	Invitrogen	L34957
Zombie UV™ Fixable Viability Kit	Biolegend	423107

Supplementary Table 2: qPCR primers

Gene	Accession number	Forward primer	Reverse primer
Acaca	NM_133360.2	CAGCTGGTGCAGAGGTACCG	TCTACTCGCAGGTACTGCCG
Acox1	NM_015729	GGGACCCACAAGCCTCTGCCA	GTGCCGTCAGGCTTCACCTGG
Acta2	NM_007392.3	AGCCATCTTTCATTGGGATGG	CCCCTGACAGGACGTTGTTA
Cidec	NM_178373	CCATCAGAACAGCGCAAGAAG	AGAGGGTTGCCTTCACGTTC
Cd36	NM_001159558	GCAAAGAACAGCAGCAAAATC	CAGTGAAGGCTCAAAGATGG
Col1a1	NM_007742.3	GAGAGGTGAACAAGGTCCCG	AAACCTCTCTCGCCTCTTGC
Cpt1a	NM_013495	AGGAGACAAGAACCCCAACA	AAGGAATGCAGGTCCACATC
Fasn	NM_007988	CACAGGCATCAATGTCAACC	TTTGGGAAGTCCTCAGCAAC
Fabp1	NM_017399.4	GCCACCATGAACTTCTCCGGCA	GGTCCTCGGGCAGACCTATTGC
Il1b	NM_008361	GACCCCAAAAGATGAAGGGCT	ATGTGCTGCTGCGAGATTTG
Il6	NM_031168.2	CCTCTCTGCAAGAGACTTCCAT	ACAGGTCTGTTGGGAGTGGT
Il23a	NM_031252.2	GCACCAGCGGGACATATGAA	CAAGCAGAACTGGCTGTTGTC
Plin4	NM_020568.3	TGCCCCCTCATCTAAAGTGTC	AGGCATCTTCACTGCTGGTC
Rplp0	NM_007475	TCTGGAGGGTGTCCGCAACG	GCCAGGACGCGCTTGTACCC
Scd1	NM_009127.4	GCTCTACACCTGCCTCTTCGGGAT	TCCAGAGGCGATGAGCCCCG
Stk11	NM_011492.4	GTGCCAAGCTCATGGGTACT	CACCGAGGTCGGAGATCTTG
Tgfb1	NM_011577	GCTGAACCAAGGAGACGGAA	ATGTCATGGATGGTGCCCAG
Timp1	NM_011593	AGTGCCTGCAGCTTCTTGGT	CAGCCAGCACTATAGGTCTTTGAG

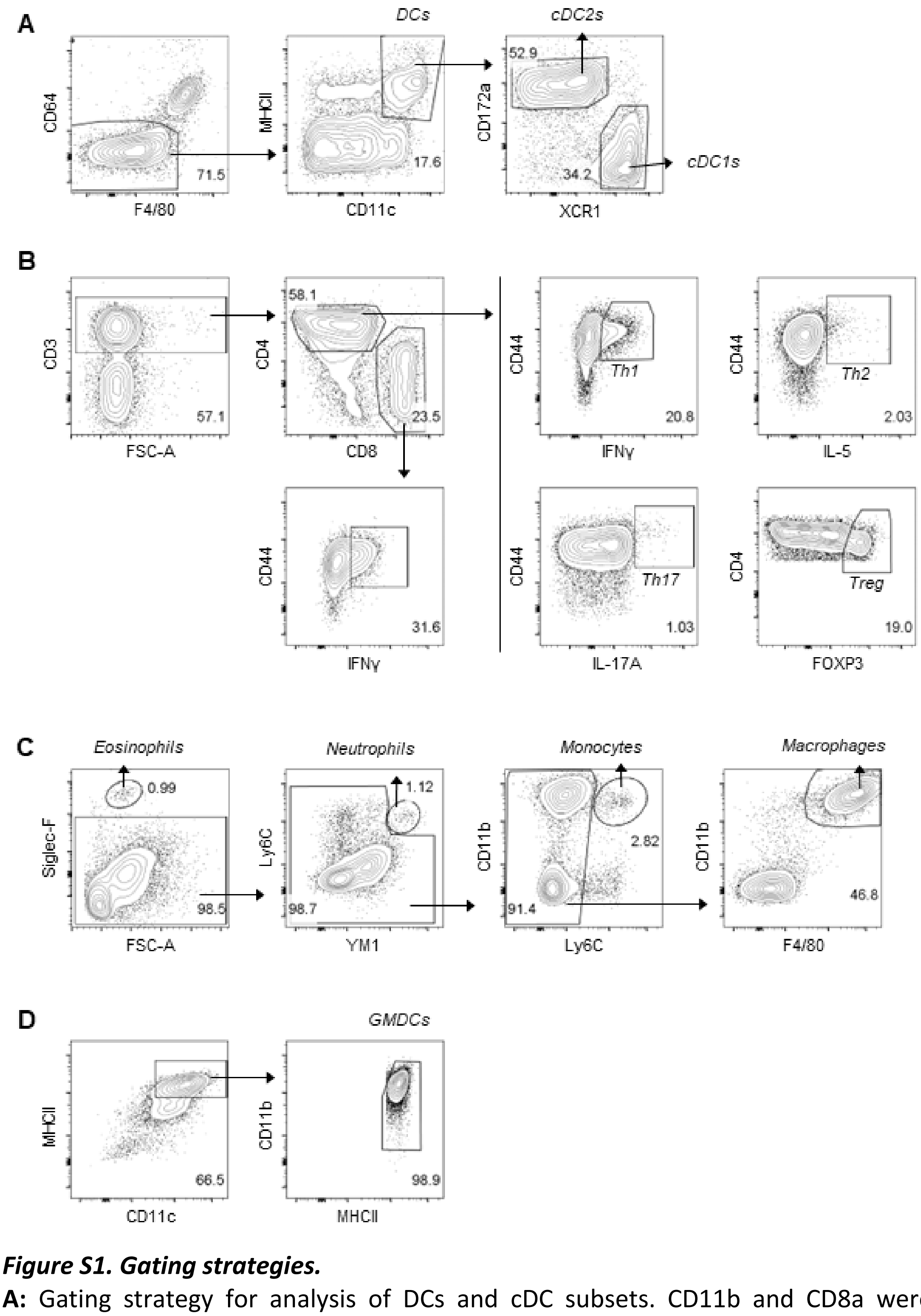

Figure S1. Gating strategies.

A: Gating strategy for analysis of DCs and cDC subsets. CD11b and CD8a were used as alternatives for CD172a and XCR1, respectively. **B:** Gating strategy for T (helper) cell subsets is shown. **C:** Gating strategy for identification of myeloid cell subsets. **D:** Gating strategy for identification of GMDCs. Isolated cells were pre-gated on live $CD45^+$ single cells. For T (helper) cell subset analysis, cells were additionally pre-gated as $lineage^-$, which included antibodies directed against B220, CD11b, CD11c, GR-1 and NK1.1. Representative sample was chosen from eWAT samples for A-C. Gating strategies were similar for the indicated cell populations in liver and spleen.

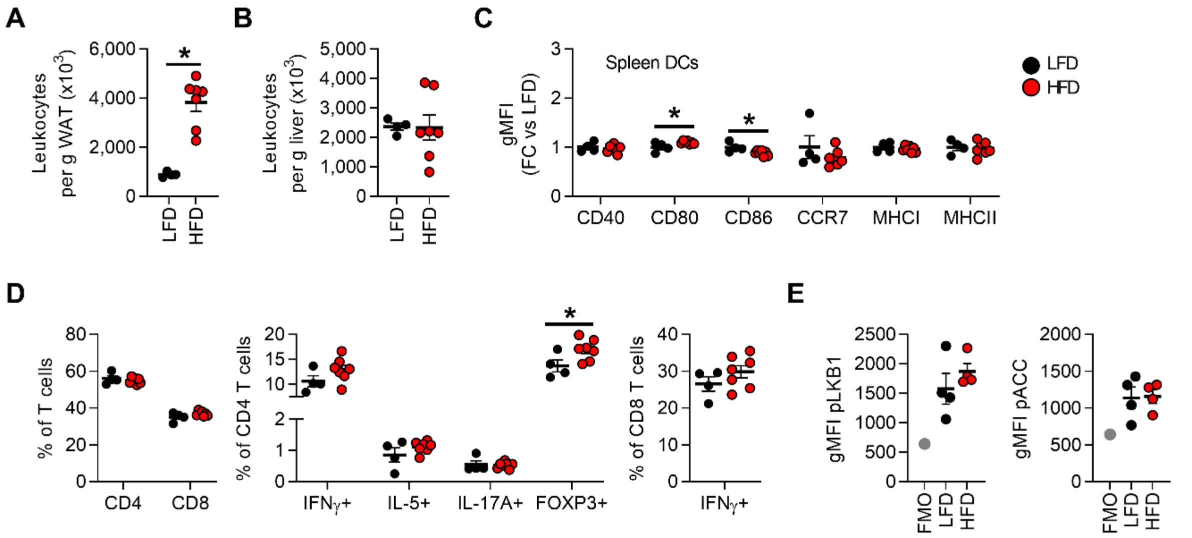

Figure S2. Leukocytes per gram WAT and liver, and splenic DCs and T cells are mostly unaffected by obesity.

Mice were fed a LFD (black symbols) or a HFD (red symbols) for 24 weeks. **A-B**: Absolute numbers of leukocytes per g tissue in eWAT (*A*) and liver (*B*). **C**: At sacrifice, spleen was collected and immune cells were isolated and analysed by flow cytometry. Relative expression of indicated DC markers by splenic DCs. **D**: Cells were restimulated with PMA/ionomycin in the presence of Brefeldin A for detection of intracellular cytokines, and were analysed by flow cytometry. CD4 and CD8 T cell, Th1, Th2, Th17 and Treg CD4 T cell, and IFNγ$^+$ CD8 T cell percentages in spleen. **E**: Spleens were immediately formaldehyde-fixed after collection and immune cells were isolated. Phosphorylated LKB1 (Ser431) and ACC (Ser79) were measured in DCs from spleen by flow cytometry. Results are expressed as means ± SEM. Statistical analyses were performed using unpaired t-tests. * P<0.05 vs LFD (n = 4-7 mice per group). Related to figure 1.

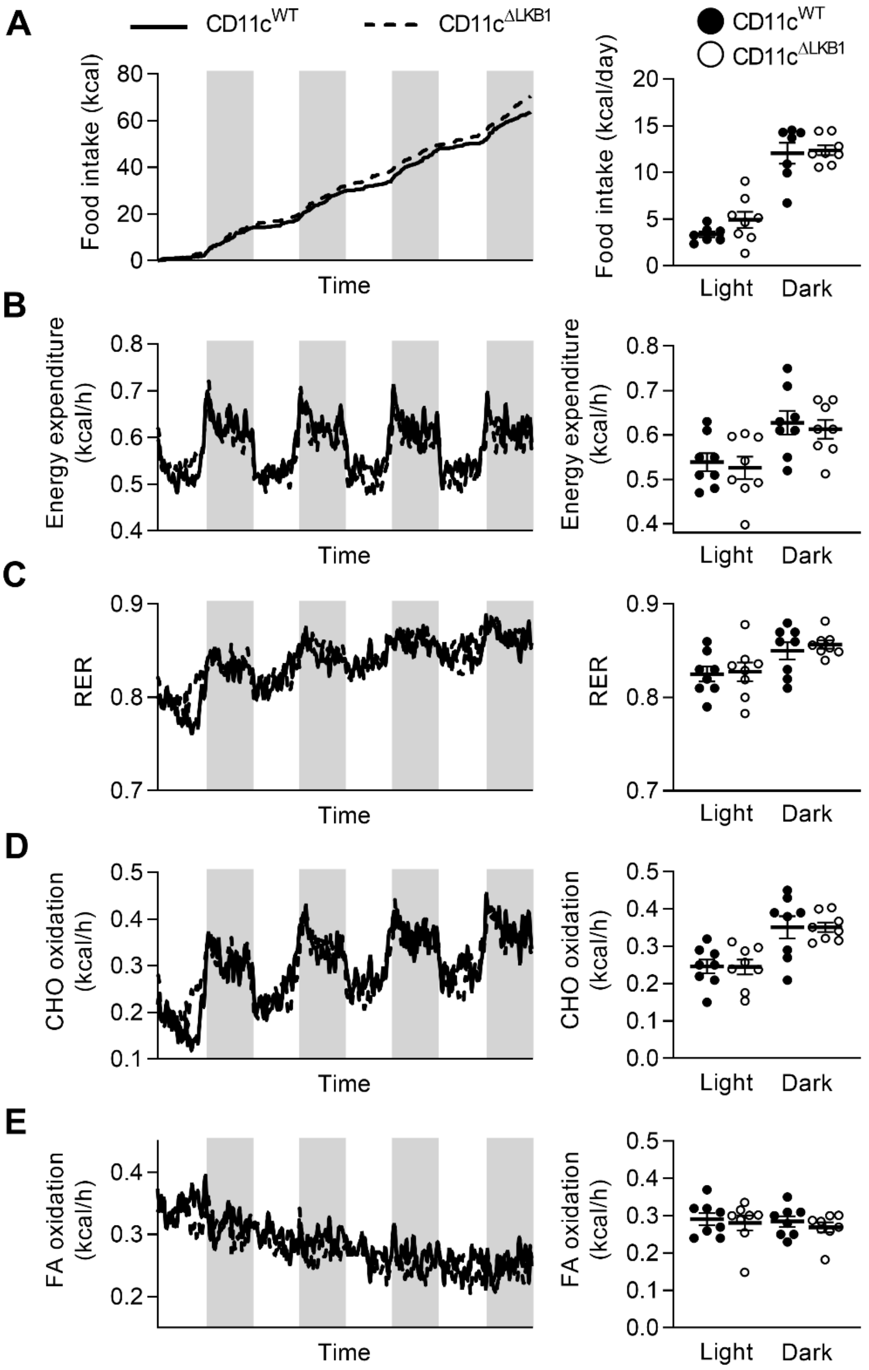

Figure S3. LKB1 deficiency in DCs does not affect food intake and whole-body energy expenditure.

CD11c^{WT} (black symbols) and CD11c$^{\Delta LKB1}$ (open symbols) mice were fed a HFD for 18 weeks. At week 15, mice were subjected to individual indirect calorimetric measurements using fully automated metabolic cages with free access to food and water. **A-E:** Cumulative food intake (*A*), energy expenditure (EE; *B*), respiratory exchange rate (RER; *C*), carbohydrate (CHO; *D*) and fatty acid (FA; *E*) oxidation were measured for 4 consecutive days (white part = light phase; grey part = dark phase). The daily averages for each of the abovementioned parameters were calculated. Results are expressed as means ± SEM. (n = 7-8 mice per group). Statistical analyses were performed using two-way ANOVA followed by Fisher's post-hoc tests. Related to figure 2.

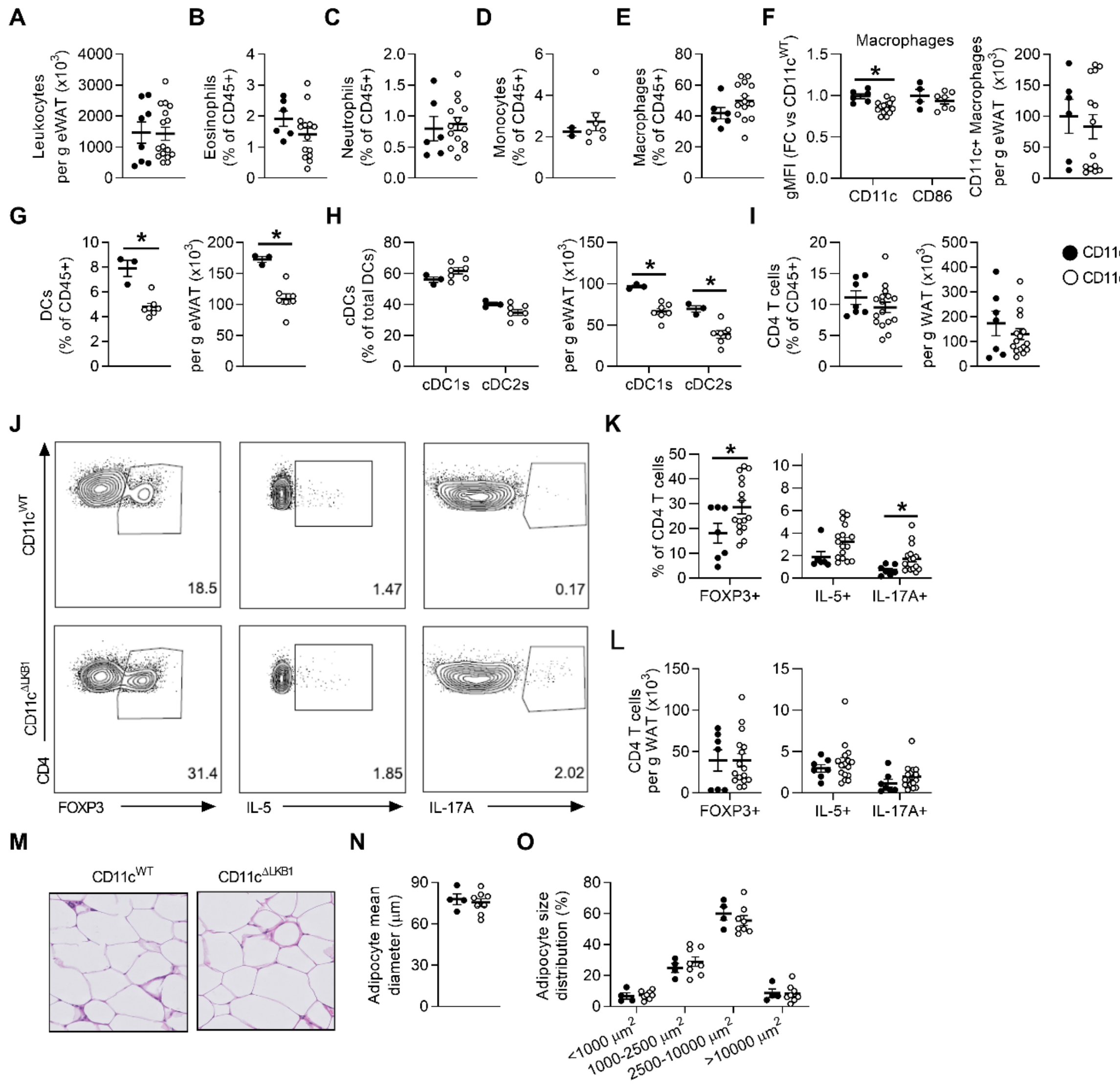

Figure S4. LKB1 deficiency in DCs does not aggravate adipose tissue immunometabolic dysfunctions in obese mice. CD11c^{WT} (black symbols) and CD11c$^{\Delta LKB1}$ mice (open symbols) were fed a HFD for 18 weeks. A-I: At sacrifice, eWAT was collected and immune cells isolated and analysed by flow cytometry. Total leukocytes per gram eWAT were quantified (A). Percentages of eosinophils (B), neutrophils (C), monocytes (D) and macrophages (E) in eWAT expressed as frequencies of total leukocytes. Expression of CD11c and CD86 on eWAT macrophages relative to CD11c^{WT} mice and total CD11c^{+} macrophages as cells per gram eWAT (F). Abundances of DCs (G), cDC subsets (H) and CD4 T cells (I). J-N: eWAT immune cells were restimulated with PMA and ionomycin in the presence of Brefeldin A for intracellular cytokine detection. Representative plots (J) and percentages of FOXP3^{+} Treg , IL-5^{+} Th2 and IL-17A^{+} Th17 cells were determined as frequencies of CD4 T cells (K) and as absolute cell number per gram eWAT (L). M: A part of eWAT was sectioned and H&E-stained. N-O: Mean adipocyte diameter (N) and adipocyte size distribution (O) were quantified from H&E stained slides. Data shown are a pool of two independent experiments, except for D, F-H and O-Q. Results are expressed as means ± SEM. Statistical analysis were performed using unpaired t-tests. * P<0.05 vs CD11c^{WT} (n = 7-16 mice per group for A-C, E and I-N; n = 3-8 mice per group for D, F-H and O-Q).

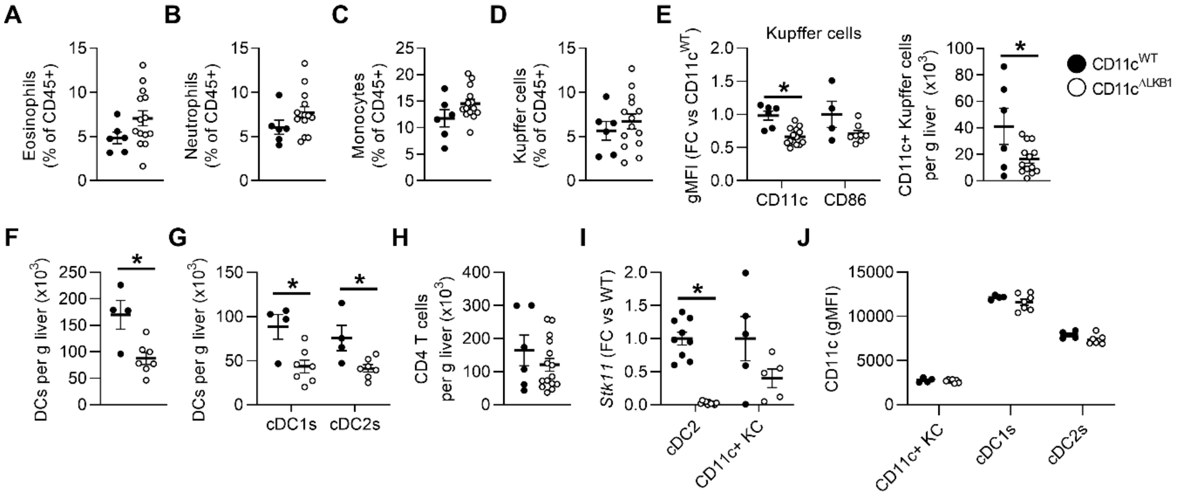

Figure S5. Effects of LKB1 deletion from DCs on myeloid cell subsets in the liver.

CD11c^{WT} (black symbols) and CD11c$^{\Delta LKB1}$ (open symbols) mice were fed a HFD for 18 weeks. At sacrifice, liver was collected and immune cells were isolated and analysed by flow cytometry. **A-D**: Percentages of hepatic eosinophils (*A*), neutrophils (*B*), monocytes (*C*) and Kupffer cells (*D*) expressed as frequencies of total leukocytes. **E:** Expression of CD11c and CD86 on Kupffer cells, expressed as fold change *vs* CD11c^{WT} and total CD11c^{+} Kupffer cells per gram liver. **F-H**: absolute cell numbers per gram liver for total cDCs (F), cDC subsets (G) and CD4 T cells (H). **I**: Expression of *Stk11* as measured by RT-qPCR in cDC2s and CD11c^{+} Kupffer cells (KC) from chow-fed mice. **J:** Expression of CD11c in CD11c^{+} Kupffer cells and dendritic cell subsets. Data shown are a pool of two independent experiments, except for CD86 expression in E, I and J. Results are expressed as means ± SEM. Statistical analyses were performed using unpaired t-tests. * P<0.05 vs CD11c^{WT} (n = 4-14 mice per group). Related to figure 3.

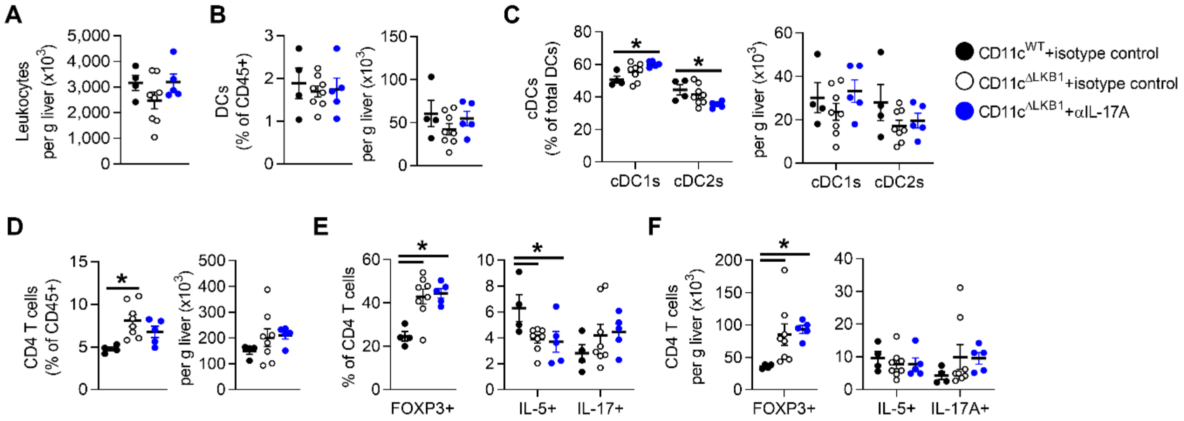

Figure S6. Effects of IL-17A neutralization on hepatic immune cells.

Mice were treated as described in the legend of figure 5. **A-D**: At sacrifice, liver was collected and immune cells were isolated and phenotyped by flow cytometry. Total number of leukocytes per gram liver (*A*), and abundances of DCs (*B*), cDC subsets (*C*) and CD4 T cells (*D*) were determined. **E-F**: Hepatic leukocytes were restimulated with PMA/ionomycin in the presence of Brefeldin A for detection of intracellular cytokines. Abundance of FOXP3^{+} Tregs (*E*), IL-5^{+} Th2 cells and IL-17A^{+} Th17 cells (*F*) were determined as frequencies of CD4 T cells and as absolute cell number per gram liver. Data shown are a pool of two independent experiments. Results are expressed as means ± SEM. Statistical analyses were performed using one-way ANOVA followed by Fisher's post-hoc tests. * P<0.05 vs CD11c^{WT} (n = 4-8 mice per group). Related to figure 4.

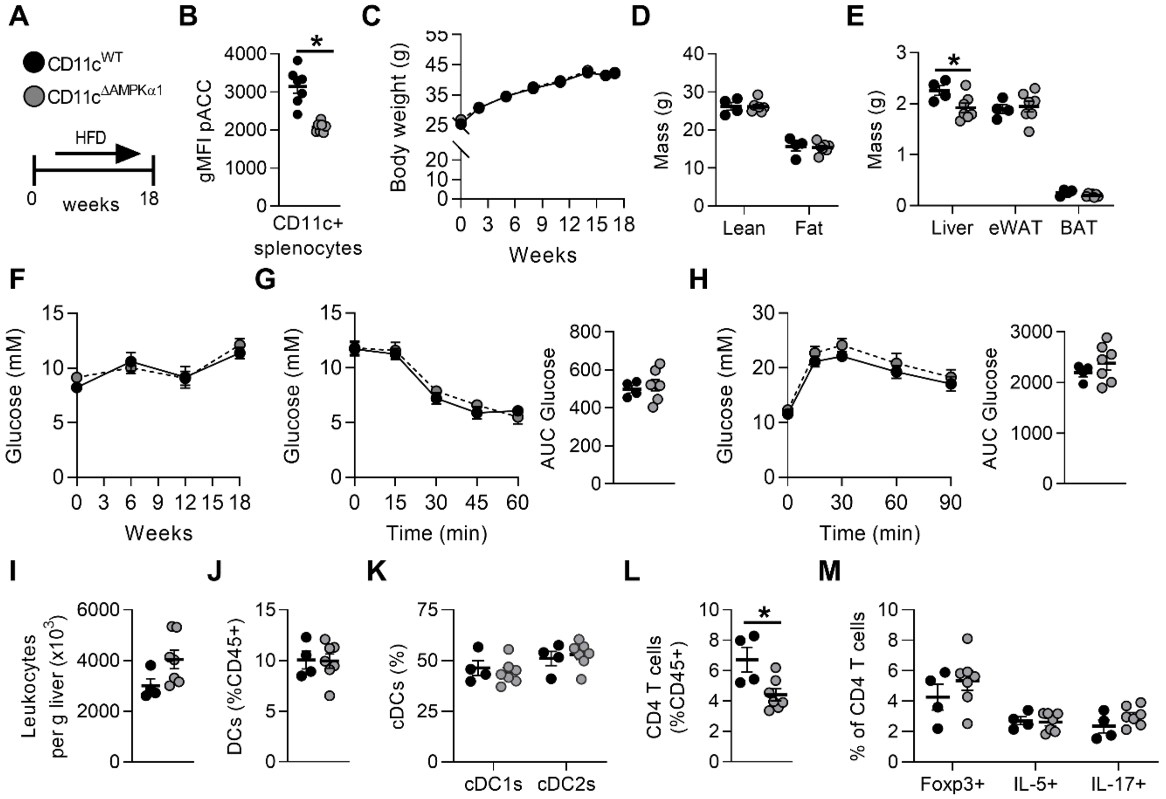

Figure S7. Deletion of AMPKα1 from DCs does not recapitulate the immunometabolic phenotype of CD11c$^{\Delta LKB1}$ mice.

A: Staining for the AMPK-specific phosphorylation site Ser79 on ACC in CD11c+ splenocytes. **B:** CD11c^{WT} (black symbols) and CD11c$^{\Delta AMPK\alpha1}$ mice (grey symbols) were fed a HFD for 18 weeks. **C**: Body weight was monitored throughout the experiment. **D-E**: Body composition (*D*) and weights of liver, eWAT and BAT (*E*) were measured at the end of the experiment. **F**: Fasting blood glucose was measured at the indicated weeks. **G**: An i.p. insulin tolerance test was performed 1 week before sacrifice and AUC calculated. **H**: An i.p. glucose tolerance test was performed 1 week before sacrifice and AUC calculated. **I-L**: At sacrifice, liver was collected and immune cells isolated. Total leukocytes per gram liver were quantified (*I*). Percentages of DCs (*J*), cDC subsets (*K*) and CD4 T cells (*L*) were determined by flow cytometry. **M**: Liver leukocytes were restimulated with PMA and ionomycin in the presence of Brefeldin A for intracellular cytokine detection. Percentages of FOXP3^{+} Treg, IL-5^{+} Th2 and IL-17A^{+} Th17 cells were determined as frequencies of CD4 T cells. Results are expressed as means ± SEM. Statistical analyses were performed using unpaired t-tests (B, D+E, G-M) or two-way ANOVA followed by Fisher's post-hoc tests (C, F-H). * P<0.05 vs CD11c^{WT} (n = 4-7 mice per group).

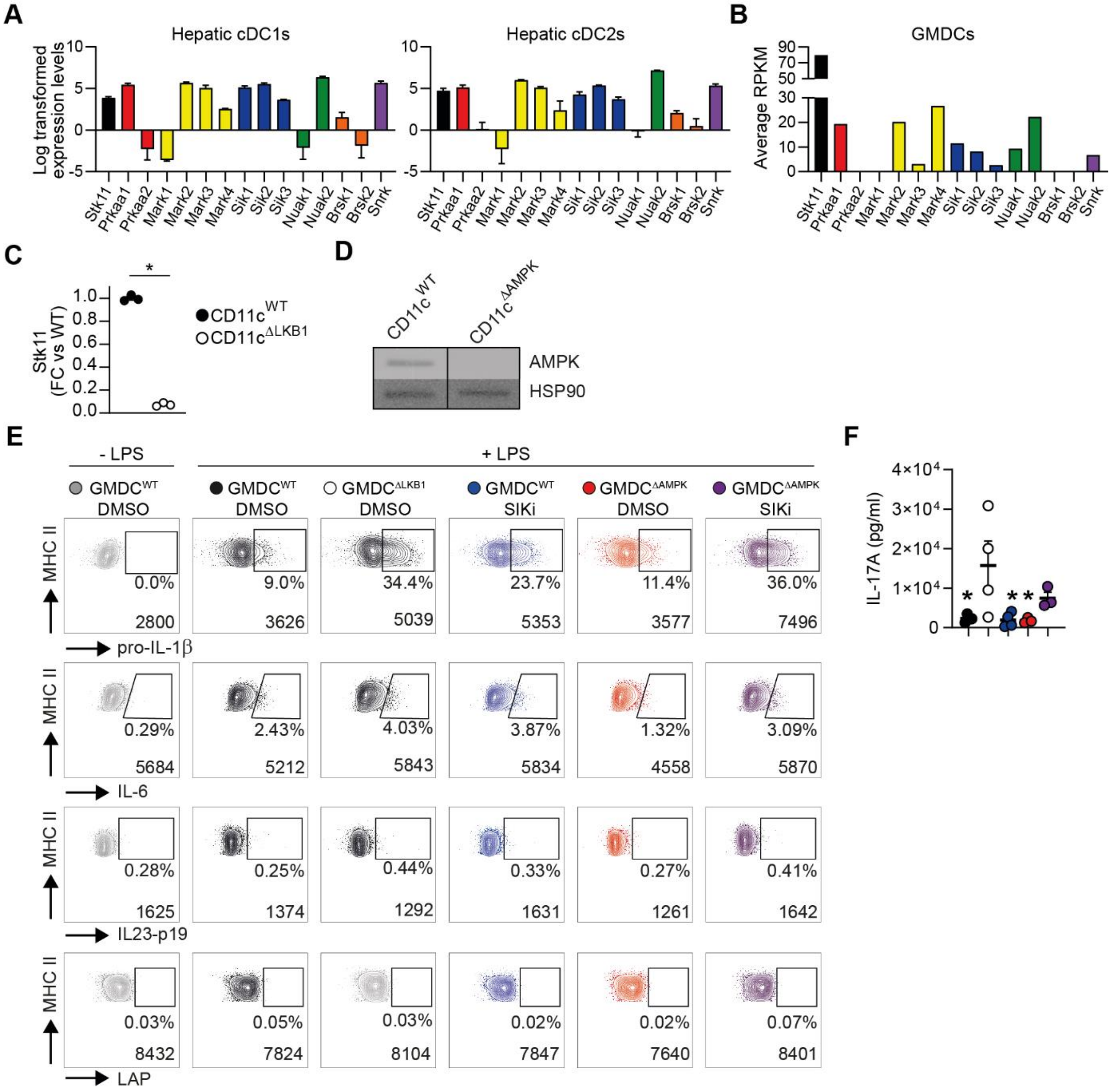

Figure S8. Transcriptional analysis of LKB1 and its substrates in DCs.

A-B: Expression of *Stk11* (encoding LKB1) and its downstream targets *Prkaa1-2* (encoding AMPKα1-2), *Mark1-4*, *Sik1-3*, *Nuak1-2*, *Brsk1-2* and *Snrk* in murine hepatic cDC1s and cDC2s (Gainullina A. et al., *bioRxiv*. 2020; *A*) and mature GM-CSF-elicited bone marrow DCs (GMDCs; Liu et al., *J. Immunol.* 2015; *B*). **C:** *Stk11* expression in GMDCs from CD11c^{WT} (black symbols) and CD11c$^{\Delta LKB1}$ mice (open symbols) at day 10 of culture. **D**: AMPK expression in GMDCs from CD11c^{WT} and CD11c$^{\Delta AMPK}$ at day 10 of culture. Lanes were run on the same gel, but were non-contiguous. Data is representative of 2 biological replicates. **E:** GMDCs were stimulated with or without LPS in the presence of Brefeldin A for intracellular cytokine detection. Representative plots for figure 5E, depicting the frequencies of positive cells and gMFI. **F:** IL-17A detected in supernatant after a 48 hour antigen-specific restimulation of popliteal lymph node cells. Results are expressed as means ± SEM. Statistical analyses were performed using unpaired t-tests (C) or one-way ANOVA followed by Dunnett's post-hoc tests (F). * $P<0.05$ vs CD11c^{WT} (C) or *$P<0.05$ vs LKB1 KO (F). Related to figure 5.
